# The major histocompatibility complex in Old World camelids and low polymorphism of its class II genes

**DOI:** 10.1186/s12864-016-2500-1

**Published:** 2016-03-01

**Authors:** Martin Plasil, Elmira Mohandesan, Robert R. Fitak, Petra Musilova, Svatava Kubickova, Pamela A. Burger, Petr Horin

**Affiliations:** Department of Animal Genetics, Veterinary and Pharmaceutical University, Brno, Czech Republic; Ceitec VFU, RG Animal Immunogenomics, Brno, Czech Republic; Research Institute of Wildlife Ecology, Vetmeduni Vienna, Vienna, Austria; Institute of Population Genetics, Vetmeduni Vienna, Vienna, Austria; Department of Biology, Duke University, Durham, NC USA; Department of Genetics and Reproduction, Veterinary Research Institute, Brno, Czech Republic

**Keywords:** MHC, Exon 2, Wild two-humped camel, Bactrian camel, Dromedary, Camelini, aDNA

## Abstract

**Background:**

The Major Histocompatibility Complex (MHC) is a genomic region containing genes with crucial roles in immune responses. MHC class I and class II genes encode antigen-presenting molecules expressed on the cell surface. To counteract the high variability of pathogens, the MHC evolved into a region of considerable heterogeneity in its organization, number and extent of polymorphism. Studies of MHCs in different model species contribute to our understanding of mechanisms of immunity, diseases and their evolution. Camels are economically important domestic animals and interesting biomodels. Three species of Old World camels have been recognized: the dromedary (*Camelus dromedarius*), Bactrian camel (*Camelus bactrianus*) and the wild camel (*Camelus ferus*). Despite their importance, little is known about the MHC genomic region, its organization and diversity in camels. The objectives of this study were to identify, map and characterize the MHC region of Old World camelids, with special attention to genetic variation at selected class MHC II loci.

**Results:**

Physical mapping located the MHC region to the chromosome 20 in Camelus dromedarius. Cytogenetic and comparative analyses of whole genome sequences showed that the order of the three major sub-regions is “Centromere - Class II – Class III – Class I”. *DRA, DRB, DQA* and *DQB* exon 2 sequences encoding the antigen binding site of the corresponding class II antigen presenting molecules showed high degree of sequence similarity and extensive allele sharing across the three species. Unexpectedly low extent of polymorphism with low numbers of alleles and haplotypes was observed in all species, despite different geographic origins of the camels analyzed. The *DRA* locus was found to be polymorphic, with three alleles shared by all three species. *DRA* and *DQA* sequences retrieved from ancient DNA samples of *Camelus dromedarius* suggested that additional polymorphism might exist.

**Conclusions:**

This study provided evidence that camels possess an MHC comparable to other mammalian species in terms of its genomic localization, organization and sequence similarity. We described ancient variation at the *DRA* locus, monomorphic in most species. The extent of molecular diversity of MHC class II genes seems to be substantially lower in Old World camels than in other mammalian species.

**Electronic supplementary material:**

The online version of this article (doi:10.1186/s12864-016-2500-1) contains supplementary material, which is available to authorized users.

## Background

Pathogens are considered to be one of the driving forces of evolution. The major histocompatibility complex (MHC) is a genomic region containing immune response (IR) genes, which play a crucial role in host and pathogen interactions. MHC class I and class II genes encode antigen-presenting molecules responsible for the dual recognition of antigenic peptides on the cell surface [1]. MHC-encoded antigen presenting molecules are thus directly involved in molecular interactions with specific peptides derived from pathogens to which a population is exposed. Therefore, the MHC genes are under strong selective pressure and contain signatures of both positive and negative selection [2]. To counteract the high variability of pathogens and pathogen-derived molecules, the MHC of Gnathostomata evolved into a region of considerable heterogeneity in its organization, number, and extent of polymorphism both within and between species [3–5]. Spanning approximately 4 megabases (Mb), the MHC region consists of hundreds of different genes with a variety of functions including antigen presentation and processing as well as non-immune processes [1]. Consequently, MHC class I and class II genes are amongst the most polymorphic genes studied in vertebrates with more than 100 alleles reported in different species, including humans [6, 7]. In MHC class II genes, a majority of the functionally important polymorphisms are concentrated in exon 2, which encodes the antigen-binding site of the molecule [8, 9]. This diversity is correlated with pathogen richness [10].

Infectious diseases of livestock have a significant economical impact on animal husbandry, and they also may affect human health directly or through food chains. Studies of MHCs in different model species contribute to our understanding of mechanisms of infectious diseases. Economically as well as culturally important among domestic animals are camels, with their long history of adaptation to arid environments and with their capability of providing transport and various commodities important for human development [11–13]. Currently, three extant species of Old World camels are recognized [14–16]. The dromedary (*Camelus dromedarius*) and Bactrian camel (*Camelus bactrianus*) are key domestic species in semi-arid and desert areas and are used for food production and camel racing throughout many Arabian, Northern African and Asian countries. The only surviving and critically endangered wild camel species (*Camelus ferus*) is closely related to them and diverged from the domestic Bactrian camel approximately 1,000,000 years ago [14, 15].

In terms of infectious disease, the Old World camels (genus *Camelus*) are an interesting biomodel. They are resistant to serious infections threatening other livestock inhabiting the same geographical regions [17, 18]. Recently, dromedaries have been identified as potential vectors of the Middle East Respiratory Syndrome (MERS) virus [19, 20]. The immune system of camels displays characteristic features of practical importance, like heavy chain antibody homodimers [21, 22]. Furthermore, all extant *Camelus* species are renowned for their ability to cope with harsh environmental challenges, including high temperatures, drought, and famine combined with high level of physical activities. However, little is known about the MHC genomic region, its organization and diversity in camels [23].

Recently, draft genome sequences have been made available for all three species [13, 16, 24, 25]. Although some MHC genes have been annotated in these assemblies, the draft genome sequences still contain gaps and errors [25]. It has been repeatedly recognized for other species, that the complexity of the MHC and other complex regions involved in mechanisms of immunity and disease cannot be resolved at this level [26]. Moreover, in camels the full genome sequences available were derived from single individuals, while the complexity of MHC and of its sub-regions should be based on targeted re-sequencing of multiple individuals originating from genetically different populations [27].

Therefore, the objectives of this study were to i) identify and map the MHC region in the genomes of Old World camelids, ii) characterize its overall genomic organization, and iii) characterize the genetic variation at selected class MHC II loci in modern and ancient samples.

## Methods

### Sample collection and DNA extraction

Peripheral blood from different populations of Mongolian Bactrian camels (*C. bactrianus*, *n* = 57) and dromedaries from Jordan (*C. dromedarius*, *n* = 31) was collected during routine veterinary procedures. DNA extractions were performed using the NucleoSpin® Blood kit (Macherey-Nagel) or the standard phenol-chloroform extraction [28]. Additionally, we acquired previously extracted DNA from different geographic populations of *C. dromedarius* (*n* = 35), *C. bactrianus* (*n* = 1), and *C. ferus* (*n* = 20) from the sample database stored at the Vetmeduni Vienna, Research Institute of Wildlife Ecology (Additional file 1). These samples represent a majority of the geographical range within each camel species, thus maximizing our ability to sample the breadth of genetic diversity. All DNA samples were stored at −20 °C prior to analysis. The numbers of individuals from each species, which were analyzed for the MHC class II genes, are presented in Table 1.

**Table 1:** Total numbers of Old World camelids analyzed for MHC class II genes

Species	DRA (n)	DRB (n)	DQA (n)	DQB (n)
*Camelus ferus*	18	11	8	5
*Camelus bactrianus*	30	42	47	18
*Camelus dromedarius*	37	54	19	7

In addition to the modern samples collected above, ancient dromedary specimens (*n* = 3) were collected from an archaeological site in Jordan (Aqaba) as part of a separate study (Mohandesan et al. submitted). The samples stem from the Mamluk and Ottoman period, ranging from 250 to 740 years ago (Table 2). The ancient dromedary specimens were prepared in a dedicated ancient DNA laboratory at the Paleogenetic Core Facility of the ArchaeoBioCenter at the Ludwig-Maximilian-University, Munich, Germany, following a range of standard contamination precautions [29]. DNA was extracted from bone material following the protocols described in Rohland and Hofreiter [30], and Rohland et al. [31]. Extractions were conducted in batches of seven samples and one extraction blank as a control.

**Table 2:** Historical dromedary camel samples used in this study

Sample ID	Site	Excavation year	Period (Dates)	Gene partially recovered
AQ30	Jordan	2006	Mamluk (1260–1456 AD)	DRA
AQ34	Jordan	2006	Mamluk (1260–1456 AD)	DRA
AQ40	Jordan	2007	Ottoman (1456–1870 AD)	DQA

### Physical mapping of the MHC region

Fluorescence in situ hybridization (FISH) probes were designed to physically map the MHC region and to establish its orientation relative to the centromere. One FISH probe specific for the class I region (MHCI) was placed on gene *TRIM39* at the scaffold KN277189.1 (positions: 996661–1006833, and a class II specific probe (MHCII) was placed on gene *FANCA* at the scaffold KN276514.1 (positions: 2132659–2136283). Both scaffolds are part of the Bactrian camel genome assembly [GenBank: JARL00000000.1]. The primers used for amplifying the FISH probes are listed in Table 3. The PCR products were cloned into the pDrive Cloning Vector (Qiagen) and the recombinant plasmids were labeled with digoxigenin-11-dUTP or biotin-16-dUTP (Roche Diagnostics GmbH, Mannheim, Germany) using the Nick Translation Reagent Kit (Vysis, Richmond, UK). The labeled probes were used for standard FISH to dromedary metaphase chromosomes prepared from peripheral blood culture [32]. Hybridization of MHCI and MHCII probes were visualized by immunodetection using fluorescein avidin (Vector Laboratories, Burlingame, CA, USA) or anti-digoxigenin-rhodamine (Roche), respectively.

**Table 3:** Primers used to amplify different MHC sequences in Old World camelids

Locus	Name	Sequence (5' → 3')	Purpose	PCR product length
DRA	DRA-EXON2-F	TGAGAATTTTGGGTTTGCTTATGGCA	Bact, drom	514 bp
	DRA-EXON2-R	CCTCTGAGCAACACGAACGTCCTTCA		
	DRA-EXON2-WC-F	TGGGTGTTTCAGCTCTTGTG	Ferus	650 bp
	DRA-EXON2-WC-R	AGATACCATGGGTGGCAAAG		
	DRA-EXON2-3-F	CCCTGGAATTCGGGTTTAAG	Preventing allele dropout	923 bp
	DRA-EXON2-3-R	GGCTGAAAAAGCAGTTGAGC		
	DRA_F1	TGATCATCCAGGCTGAGTTC	Ancient samples	139 bp
	DRA_R1	GCAAACCGTCCAAATTCTTC		
	DRA_F2	CGTGGATCTGGAAAAGAAGG	Ancient samples	154 bp
	DRA_R2	ATTGGTGTTCGGGGTGTG		
DRB	DRB-EXON2-2-F	AGCAGTGGGGGTCCTAGTG	Bact, drom	457 bp
	DRB-EXON2-2-R	ACCCACCCGGACTCAGTATC		
	DRB-EXON2-WC-F	TTCAGGAGGAGGTGGTGATG	Ferus	764 bp
	DRB-EXON2-WC-R	CTCAGACCCCAGACCCATT		
	DRB-EXON2-3-F	GCCAGCCCTAGGCAAGTAAG	Preventing allele dropout	852 bp
	DRB-EXON2-3-R	GTTCTCTCAGACCCCAGACC		
DQA	DQA1-EXON2-F	CATGAAAGTCAATTTATCCTGTCAC	Bact	351 bp
	DQA-EXON2-2-R	AGTGAGGCCTGGTATGAAGG		
	DQA-EXON2-WC-F	ACGTGCTGGGAATTTTGTCT	Drom	414 bp
	DQA-EXON2-2-R	AGTGAGGCCTGGTATGAAGG		
	DQA-EXON2-WC-F	ACGTGCTGGGAATTTTGTCT	Ferus	646 bp
	DQA-EXON2-WC-R	GCTATGGGAGCTTTCCTTGA		
	DQA-EXON2-4-F	ATGGTGCAGAGAGCAGAAGG	Preventing allele dropout	1096 bp
	DQA-EXON2-4-R	TGGGAAACACAGTCACCTCA		
	DQA_F1	YGGCRTAAATGTCTACCAGTC	Ancient samples	133 bp
	DQA_R1	TSRAAACTTGSTAAACAGAGG		
	DQA_F2	CGTGGACTTGGAGAAGAAGG	Ancient samples	145 bp
	DQA_R2	AGCAGTAGAGTTGGAGCGTTT		
DQB	GH 28	CTCGGATCCGCATGTGCTACTTCACCAACG	Bact, drom, ferus	181 bp
	GH 29	GAGCTGCAGGTAGTTGTGTCTGCACAC		
FANCA	KN276514-F	TCAAGTCAGAAACAAGAACACAGA	FISH probe MHCII	3574 bp
	KN276514-R	CATCATTGCCAGATCCTTCA		
TRIM39	KN277189-F	CCACCCACCATGTGTATGAA	FISH probe MHCI	3459 bp
	KN277189-R	GCTGAGAGTGCAGGGATAGG		

Bact - Primer pair used to amplify samples of *Camelus bactrianus*. Drom - Primer pair used to amplify samples of *Camelus dromedarius*. Ferus - Primer pair used to amplify samples of *Camelus ferus*

### Overall genomic organization of the MHC region in Old World camelids

One of the goals in this study was to assess the order of the three major MHC sub-regions, *i.e.* class I, II and III. Recently sequenced genomes of domestic Bactrian and dromedary camels [13, 25] were analyzed to decipher the overall organization of MHC region in camels. For this purpose, class-specific but adjacent sequences located at the boundaries between the class I, II and III regions and likely to be located within the same contigs were identified in the assembled reference bovine genome Btau3.5 (Table 4). A standard BLAST search [33] of all camelid genomic resources available was then performed by using these sequences to assess their physical proximity in the (fragmented) camel genomes.

**Table 4:** Locations of BLAST hits on the Bactrian genome scaffolds KN276514.1 and KN277189.1 (Accession number JARL00000000.1)

Gene (cattle)	Scaffold	Start	End	ID	Boundaries
DQB exon 2	KN276514.1	4092056	4091824	S43263.1|53-319	MHC class II
butyrophilin-like protein 1-like		4159484	4168470	LOC504295	MHC class II scaffold
butyrophilin subfamily 1 member A1	KN277189.1	226	1044	LOC615223	MHC class I & III scaffold
NOTCH4		17978	41342	536128	MHC class III
BAT1		513067	522625	540191	MHC class III
BoLA, MHC class I A		1067171	1157210	533050	MHC class I

### Amplification and sequencing of MHC class II genes from modern camels

Due to their functional importance, we specifically focused on the analysis of the exon 2 coding sequences of four genes, namely *DRA, DRB, DQA* and *DQB.* Camel-specific primers were designed using the Primer3 software [34]. For this purpose, species- and locus-specific regions were identified by BLAST [33] search of bovine *DRA, DRB*, *DQA,* and *DQB,* exon 2 sequences against the wild Bactrian camel draft genome assembly [16]. This approach was successful for all loci except *DQB,* because no *DQB-like* sequences were found in the draft genomes available. In a second step, based on the camel-specific sequences retrieved during the first round of amplifications, primers located in the neighboring introns and amplifying the full-length exon 2 sequences could be designed. In addition, we developed a set of primers specific for each locus separately to check possible allelic dropouts (Table 3). As for *DQB*, attempts to use bovine primers DQB-LA40, DQB-LA41 and DQB-LA48 [35] failed. Eventually, the zoo-primers GH28 and GH29 amplifying *DQB* exon 2 in various mammalian species were used successfully [36]. All primer sequences and resulting PCR product lengths are summarized in Table 3.

The PCR reactions were performed in a reaction volume of 12.5 μl containing 50 μg/ml of DNA, 1x KAPA2G Buffer A (with MgCl_2_), 1x KAPA Enhancer 1, 0.2 mM of each dNTPs, 0.5 μM of forward and reverse primer and 0.5 U of KAPA2G Robust HotStart DNA Polymerase (Kapa Biosystems, USA). Negative controls were included in each PCR. Amplified PCR products were purified with ExoSAP-IT using standard protocol (Affymetrix, USA) and subjected to Sanger sequencing (Macrogen Europe, the Netherlands). Next generation sequencing (NGS) was used for a subset of samples (*n* = 20) as a part of our task to have all variants confirmed based on at least two independent PCRs. For the remaining samples, Sanger data available allowed the confirmation. Two platforms, Roche GS Junior or Illumina MiSeq were used according to standard protocols.

Data from Roche GS Junior were converted from sff to fasta format using sff_extract 0.3.0 [37]. Individual libraries within.sff file were isolated using sfffile command included in the Roche Data Analysis V2.9 [38]. Data from GS Junior and MiSeq were checked using FastQC quality control tool [39]. All reads were trimmed with QTrim v1.1 [40] and quality filtered with –q20 using cutadapt_v1.4.1 [41]. Trimmed reads were aligned to the wild camel genome reference sequence [16] [Genbank: AGVR00000000] using BWA v0.6.2 [42] with default parameters. We identified polymorphisms using Samtools v1.2 [43] and viewed results in IGViewer 2.3 [44]. For further analysis of polymorphisms, the GATK UnifiedGenotyper v2.7-2 [45] was used with the following settings [46]: sample ploidy = 40, site quality prior = 20, standard min confidence threshold for calling = 30, minimum power threshold for calling = 0.95.

The polymorphisms identified from the NGS data were validated by cloning and Sanger sequencing. Amplicons from selected heterozygous individuals were ligated to the pJET1.2/blunt vector and transformed using the CloneJet™ PCR cloning kit (Fermentas) according to the manufacturer’s instructions. *Escherichia coli* TOP 10 were used as competent cells. Colonies were screened for inserts of the expected length (~200 – 800 bp) by PCR, using the amplicon-specific primers. Purified PCR products were Sanger sequenced (Macrogen Europe). BioEdit 7.1.3.0 with implemented ClustalW algorithm for multiple alignments was used for the quality-check of the generated data using the ABI 3730XL DNA Analyzer [47].

### Amplification and sequencing of MHC class II genes from ancient dromedary samples

Exons 2 of two MHC genes, *DRA* (246 bp) and *DQA* (249 bp) from the ancient dromedary specimens were analyzed. Two overlapping regions (fragments of 138 bp and 237 bp) of each gene were amplified and sequenced (Table 2). The PCR amplification was carried out in 20 μl volume containing 1x PCR buffer (Invitrogen), 4 mM MgCl_2_ (Invitrogen), 1 mg/ml BSA (Invitrogen), 250 μM mix dNTPs (Invitrogen), 1.5 μM for each primer (Invitrogen), 0.5 U of AmpliTaq Gold (Invitrogen) and 5 μl DNA template. The PCR reactions were amplified using an iCycler™ Thermal cycler (Bio-RAD) located in a separate facility. The amplification programme consisted of initial denaturation at 94 °C for 9 min followed by 60 cycles (94 °C for 20 s, 55 °C for 30 s, 72 °C for 30 s) and a final extension of 72 °C for 4 min. The PCR products were purified using the QIAquick PCR purification kit (Qiagen) and sequenced in both directions on ABI 3730 XL-Analyzer (Eurofins MWG GmbH, Ebersberg, Germany). Each sequence position was determined from two independent PCR amplifications in both directions to avoid sequence errors caused by template damage. The MHC sequences obtained from the ancient samples were aligned to the MHC *DQA* exon 2 [GenBank: AGVR01020882.1|:14962–15210] and *DRA* exon 2 [GenBank: AGVR01020883.1|:30918–31163] reference sequences using CodonCode Aligner v.3.7.1.2 (Codon Code Corporation, USA) and compared to the NCBI nucleotide database sequences, using the BLAST [33] with default blastn parameters.

### Cross-validation of MHC class II alleles with genome re-sequencing data of dromedaries

To further validate the dromedary alleles for the MHC class II genes *DRA, DRB*, *DQA* and *DQB,* all Sanger sequences retrieved were compared to whole genome sequence (WGS) data obtained from a genome re-sequencing project of nine dromedaries (Fitak et al. in prep). Briefly, all sequencing was performed using an Illumina HiSeq and reads were trimmed and aligned to the dromedary reference [GenBank: GCA_000803125.1] [25]. We realigned reads near insertion-deletion polymorphisms, recalibrated the base quality scores, and identified variants according to the recommended guidelines for the Genome Analysis Toolkit v3.1-1 [46, 48]. We identified the genomic location of the MHC class II genes in the dromedary reference using a nucleotide BLAST [33] search. We compared these regions with the variants identified above and inferred haplotypes when phase could be unambiguously determined (≤1 heterozygous genotype in the locus). Frequency of heterozygotes was calculated based on Sanger sequences as a proportion of heterozygotes out of all individual camels sequenced.

### Phylogenetic analysis

BioEdit 7.1.3.0 with ClustalW algorithm was used to align the allelic sequences obtained [47]. The Jukes-Cantor [49] and Kimura-2-parameter [50] were chosen as best-fit evolutionary models based on the Akaike Information Criterion with correction for small sample size [51] to build distance matrices and to construct neighbor-joining phylogenetic trees [52] using MEGA6 [53]. *Vicugna pacos DRA* exon 2 [GenBank: ABRR02040968.1|:530–775], *DRB* exon 2 [GenBank: ABRR02040966.1|:2004–2273] and *DQA* exon 2 [GenBank: ABRR02040956.1|:13508–13756] sequences were used as outgroups. We assessed the nodal support in the phylogenetic tree using 1000 bootstrap replications.

General recommendations by Klein et al. [54] were followed for the designation of the camel MHC loci, *Mhc Cafe* (*C. ferus*), *Mhc Caba* (*C. bactrianus*) and *Mhc Cadr* (*C. dromedarius*), respectively. Allele numbers followed the region and locus abbreviation (e.g. MhcCafe-DRA*01). For alleles that could not be assigned to a specific locus, two-digit numbers after the asterisk symbol (*) were used. The accession numbers for alleles are GenBank: KT936396-KT936421.

## Results

### Physical mapping of the MHC region

**Fig. 1:**
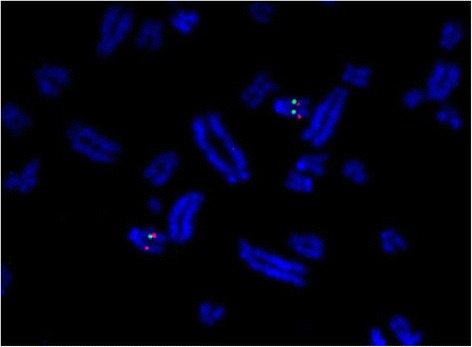
FISH MHC probes on dromedary chromosomes. Partial metaphase spread after hybridization of the probe MHCI (red) and MHCII (green) of dromedary sample. Chromosomes were counterstained with DAPI

The two FISH probes targeting the two MHC regions mapped to dromedary chromosome 20q12 according to the dromedary idiogram published by Balmus et al. [55]. It also showed that the class II region is closer to the centromere and the class I region more distant (Fig. 1).

### Overall organization of the MHC region in domestic Bactrian camels

**Fig. 2:**
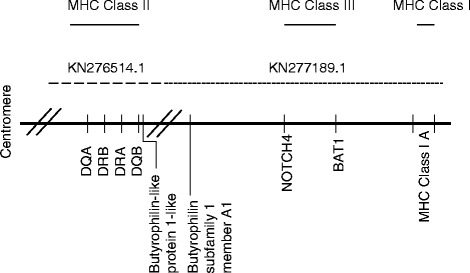
Schematic view of MHC region. Bactrian scaffolds KN276514.1 and KN277189.1 from the WGS project JARL00000000.1 were used. The // symbol indicates the end of scaffolds

The class II region containing the *DRA, DRB, DQA,* and *DQB* genes was found to be located at the end of the Bactrian genome scaffold KN276514.1 between positions 3993442 and 4091824 (see Table 4). The end of this scaffold contains the *butyrophilin like subfamily 1 member A1* associated with the MHC class II in other mammalian species [56]. Another butyrophilin-like gene was found on scaffold KN277189.1 (GenBank), physically linked to the MHC class III region genes *NOTCH4* and *BAT1*. At the end of this scaffold, the MHC class I locus A sequence was identified. These data provide information on the adjacency of both MHC class I and II regions with MHC class III loci. Based on these sequence analyses, the overall genomic organization of the Bactrian camel MHC is suggested as displayed in Fig. 2.

### Exon 2 polymorphisms of MHC class II genes in Old World camelids

**Fig. 3:**
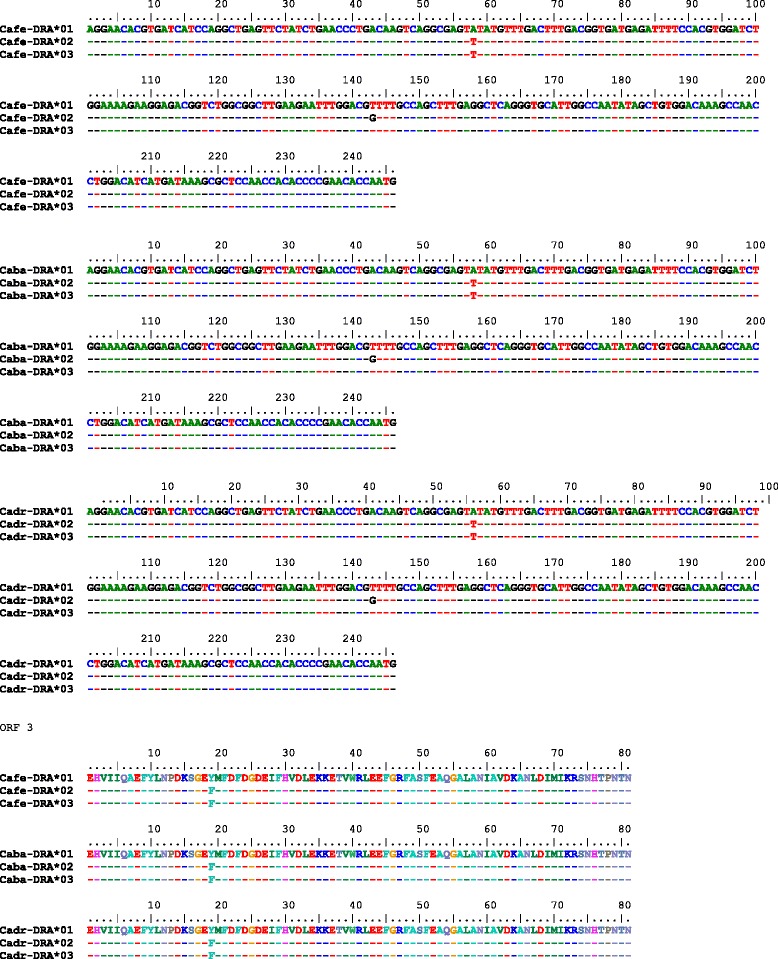
*DRA* exon 2 alleles found in *Camelus ferus* (*Cafe*), *Camelus bactrianus* (*Caba*) and *Camelus dromedarius* (*Cadr*): nucleotide sequences and *in silico* translation

**Fig. 4:**
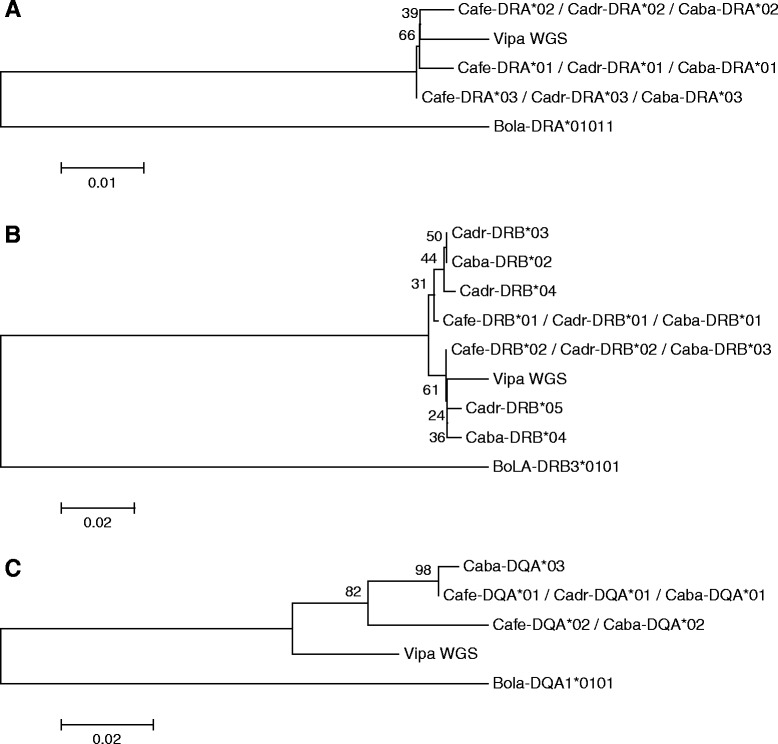
Evolutionary trees of **a** *DRA* exon 2 (sum of branch length = 0.12597149), **b** DRB exon 2 (sum of branch length = 0.28248445) and **c** DQA exon 2 in camelids (sum of branch length = 0.25550596). Bootstrap support is shown for each node as a percentage based upon 1000 replicates

*DRA exon 2*. The exon 2 of the *DRA* gene spans 246 bp in all three species. The exon contains two single nucleotide polymorphisms (SNPs), one synonymous and one non-synonymous, shared by the three species (Fig. 3). The combination of the two SNPs produced three different alleles shared again by all three species (Fig. 3). The frequencies of heterozygotes were 0.61 for wild camels, 0.53 for Bactrian camels and 0.32 for dromedaries. These differences were not statistically significant at *p* = 0.08 (chi-square 5.05). The distribution of the alleles in the groups analyzed is shown in Table 5. The corresponding phylogenetic tree is displayed in Fig. 4a. Selected chromatograms are available in Additional file 2.

**Table 5:** Distribution of MHC class II exon 2 allelic sequences in the three camel species observed in Sanger sequenced samples

		DRA alleles				DRB alleles						DQA alleles		
	(n)	*01	*02	*03	(n)	*01	*02	*03	*04	*05	(n)	*01	*02	*03
*Cafe*	18	0.306	0.25	0.444	11	0.409	0.591	-	-		8	0.875	0.125	-
*Caba*	30	0.433	0.517	0.05	42	0.702	0.048	0.048	0.202		47	0.17	0.809	0.021
*Cadr*	37	0.183	0.75	0.067	54	0.296	0.463	0.13	0.102	0.009	19	1	-	-

**Fig. 5:**
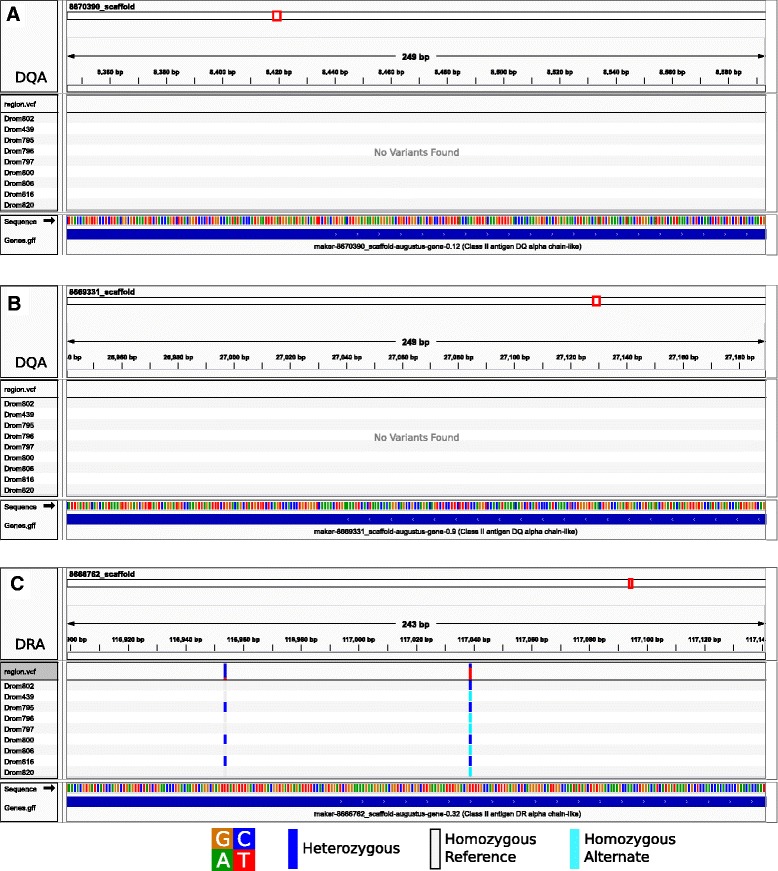
Variants identified from the genome re-sequencing of nine dromedaries in the **a**/**b** *DQA* gene exon 2, and **c** *DRA* gene exon 2. No results for the *DQB* and *DRB* genes are shown because no matches could be identified (*DQB*) or because no sequence reads were unambiguously aligned to a single region (*DRB*). The red box in the top section of each panel indicates the position along the scaffold. The bottom section of each panel shows the bases and gene annotated in the region. Images were created using IGV v2.3 (Robinson et al. 2011; Thorvaldsdóttir et al. 2013). All variants were identical to those identified using Sanger sequencing

We successfully amplified and sequenced two partial *DRA* exon 2 sequences from three ancient dromedaries (AQ30, AQ34; Table 2). The sequence recovered from AQ34 was identical to Cadr-DRA*02, while AQ30 showed an additional three C/T substitutions when compared with the reference sequence (Fig. 5).

#### DRB exon 2

**Fig. 6:**
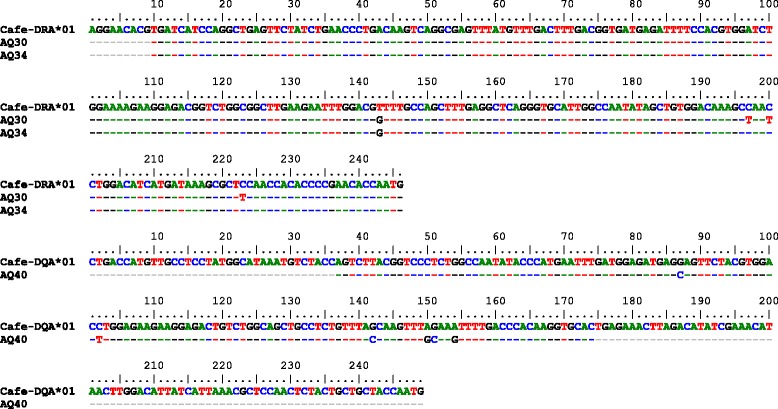
Partial aDNA sequences of *DRA* and *DQA* exons 2 from early domesticated dromedaries

The *DRB* exon 2 spans 270 bp and contains five polymorphic sites across the three species. While *C. ferus* harbors two synonymous variants (Fig. 6), two additional variants were found in *C. bactrianus*. A single non-synonymous substitution (arginine to tryptophan) was found at nucleotide position 264. Combined with *C. ferus*, four alleles were identified (Fig. 6). The distribution of the alleles among species is shown in Table 5. The frequencies of heterozygotes were 0.27 for wild camels, 0.48 for Bactrian camels and 0.59 for dromedaries. The differences were not statistically significant (*p* = 0.13; chi-square 4.14). The respective phylogenetic tree is presented in Fig. 4b. Due to reads that could not be unambiguously aligned to a single region (two matching copies of *DRB* were found, Table 6), SNPs in the *DRB* exon 2 could not be validated like in the other exons analyzed. Selected chromatograms are available in Additional file 3.

**Table 6:** Location of MHC Class II genes in the dromedary reference genome (Accession GCA_000803125.1) and the number of SNPs identified from Sanger (S_SANGER_) and next-generation (S_NGS_) sequencing

Gene	Scaffold	Start	End	Match %	S_SANGER_	S_NGS_
DRA Exon 2	8666762_scaffold	116899	117141	100	2	2
DRB Exon 2	8670257_scaffold	1687^a^	1418^a^	99.6	4	N/A
	8666762_scaffold	128830^a^	128561^a^	99.6		N/A
DQA Exon 2	8670390_scaffold	8345	8593	96.4	0	0
	8669331_scaffold	27189^a^	26941^a^	82.3		0
DQB Exon 2^b^	No significant matches detected				10	N/A

^a^reverse (3’ – 5’) orientation. ^b^DQB is annotated on the same scaffold as the highest scoring DQA match (8670390_scaffold) but no significant BLAST matches to exon 2 were detected

#### DQA exon 2

**Fig. 7:**
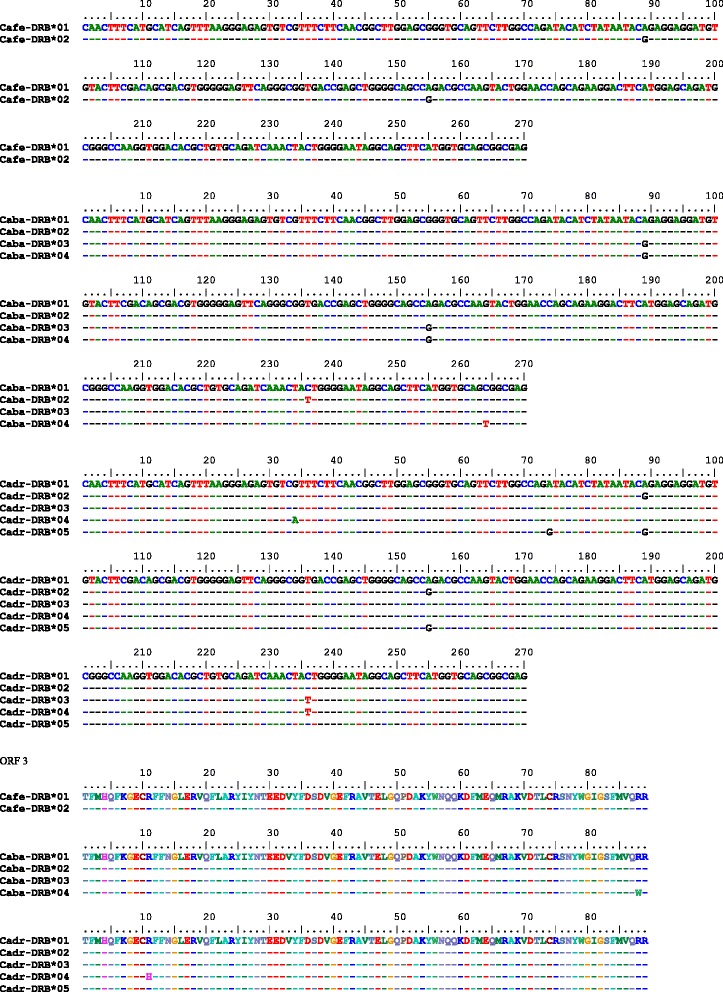
*DRB* exon 2 alleles found in *Camelus ferus* (Cafe), *Camelus bactrianus* (Caba) and *Camelus dromedarius* (Cadr): genomic nucleotide sequences and their *in silico* translation

**Fig. 8:**
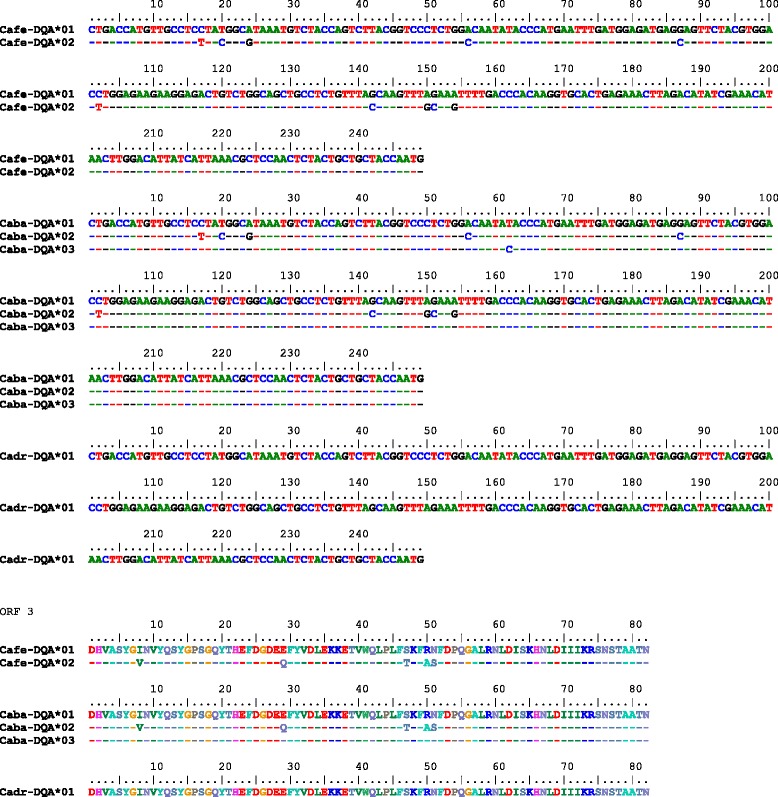
*DQA* exon 2 alleles found in *Camelus ferus* (*Cafe*), *Camelus bactrianus* (*Caba*) and *Camelus dromedarius* (*Cadr*): genomic nucleotide sequences and their *in silico* translation

Eleven polymorphic sites were observed in the *DQA* exon 2 of *C. bactrianus*, nine of them also in *C. ferus*. Four SNPs were synonymous substitutions (Fig. 7). Three haplotypes (alleles) were identified (Fig. 7), one of them shared among the three species and another one shared between wild and Bactrian camels. The distribution of alleles among species is shown in Table 5. The frequencies of heterozygotes were 0.25 for wild camels, 0.09 for Bactrian camels and 0.00 for dromedaries. The differences were not statistically significant (*p* = 0.09; chi-square 4.75). The phylogenetic tree is shown in Fig. 4c. By using *in silico* analysis of the dromedary NGS data, no variation was identified in *DQA* exon 2, which is consistent with observations from Sanger sequencing (Table 6, Figure 8b/c). The partial *DQA* exon 2 sequence retrieved from the historical sample AQ40 was identical to the *DQA* exon 2 sequence found in modern dromedaries. Selected chromatograms are available in Additional file 4.

#### DQB exon 2

**Fig. 9:**
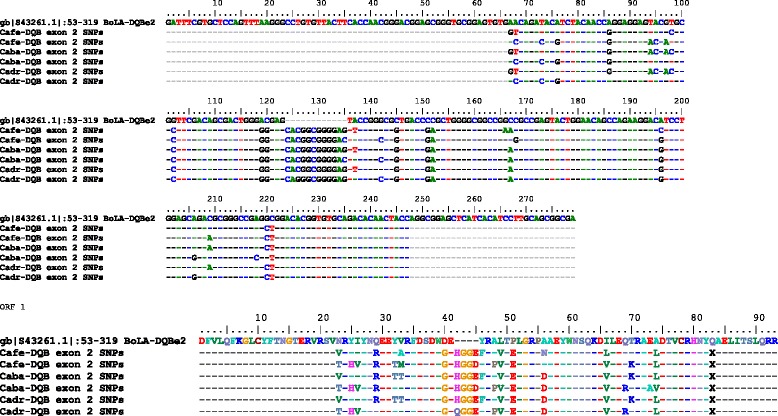
SNPs identified in the partial sequence of *DQB* exon 2 of *Camelus ferus (Cafe)*, *Camelus bactrianus (Caba)* and *Camelus dromedarius (Cadr)* and their *in silico* translation

Due to the location of the primers located within the exon 2, the sequence analyzed was truncated and contained 181 bp. A 12 bp long insertion, as compared to the corresponding bovine sequence (266 bp) [GenBank: S43261.1|:53–319] (Fig. 9) was detected in all three species. Twenty-one polymorphic sites were observed within this sequence across all three species, resulting in altogether 16 non-synonymous substitutions as shown in Fig. 9. The *C. ferus* sequences revealed a total of 16 SNPs, resulting in 11 non-synonymous substitutions. Fifteen SNPs were observed in the *DQB* exon 2 of *C. bactrianus*. Among these 15 SNPs, 11 were non-synonymous substitutions. In *C. dromedarius*, 14 SNPs with 11 non-synonymous substitutions were found (Fig. 9). Selected chromatograms are available in Additional file 5.

## Discussion

In this study we combined cytogenetics, Sanger sequencing, and NGS to investigate the MHC in Old World camels. Our results indicated that the MHC region of these three species is structurally similar to that of New World camelids (llamas) and of bovids. These results are in agreement with data previously reported for llamas [32, 57]. A combination of different techniques was necessary to recapitulate the MHC organization. We have shown that even whole genome sequences with coverage 65x could not provide complete information on the MHC region in camelids and confirmed that targeted re-sequencing is needed to resolve the complexity of this region. New techniques, like long-read NGS, are needed to further characterize this important genomic region.

FISH analysis located the MHC region on the short arm of chromosome 20 in dromedaries. Taking into consideration the high similarities between camelid genomes and karyotypes in general, we may assume that chromosomes Cba 20 and Cfe 20 carry the MHC in the other two species as well. The structure of the entire chromosome is evolutionary conserved between camelids and bovids and these chromosomes are homologous to the MHC-carrying llama and bovine chromosomes LPA20 and Bta 23, respectively [32, 55]. The MHC of camels is similar to the MHC of cattle and other bovids not only in terms of their physical location, but also of their genetic structure. The order and orientation of the three major regions, *i.e.* centromere – class II – class III and class I as inferred from the WGS [25] were directly visualized by the FISH (Fig. 1).

A general comparison between the three camel species showed substantial similarity and shared features among their MHCs. They seem to reflect similarities of their genomes in general rather than to be specific for the MHC region. Comparisons of mitochondrial genomes revealed 99 % sequence identity between *C. ferus* and *C. bactrianus*, and 93 % identity between *C. bactrianus* and *C. dromedarius* (Mohandesan et al. in prep). High number of shared alleles as well as the overall low allele count as shown in the Fig. 4 for respective loci suggests that the conservation of these sequences among camelids is still higher than similarities with other species and among these other species as shown in Table 7. Sequence IDs used for comparison in Table 7 are listed in Table  8. In camels, the *DRA* locus seems to be the most conserved, *DRB* the most divergent, with *DQA* intermediate.

**Table 7:** Overall exon 2 similarities of nucleotide sequences (n) and peptide sequences (p) between camel, cattle, human, pig, horse, sheep and goat. Due to high similarity between all camels only *Camelus ferus* sequences were used as reference Accession numbers are listed in Table 8

	DRA		DRB		DQA	
	(n)	(p)	(n)	(p)	(n)	(p)
cattle	0.894	0.84	0.785	0.652	0.835	0.768
human	0.919	0.901	0.77	0.674	0.843	0.78
pig	0.939	0.901	0.756	0.64	0.835	0.805
horse	0.923	0.864	0.752	0.573	0.819	0.756
sheep	0.77	0.852	0.732	0.607	0.827	0.768
goat	0.774	0.864	0.766	0.629	0.835	0.78

**Table 8:** Accession numbers of MHC class II exon 2 sequences used for comparison in Table 7

	DRA	DRB	DQA
camel	gb|AGVR01020883.1|:30918–31163	gb|AGVR01020883.1|:19231–19500	gb|AGVR01020882.1|:14962–15210
cattle	gb|DQ821713.1|:48–293	gi|21668455:285–554	gi|116078064:75–316
human	gi|301171411:192–437	gi|262072349|gb|GU066757.1|	gb|L34082.1|HUMMHDQAAA:14–262
pig	gb|EU722917.1|:103–348	gb|GU263817.1|:372–641	gi|166065110|gb|EU195146.1|
horse	gb|JN035629.1|:26–271	gb|JN035622.1|:267–536	gb|JQ254060.1|:84–332
sheep	gi|220981783:85–327	gb|AH001247.2|SEG_SHPMHDQB0:204–473	gb|M33304.1|SHPMHDQAA:359–607
goat	gi|2618610:2117–2362	gi|2575824|dbj|AB008347.1|	gb|AY464656.1|:96–344

From the evolutionary perspective, only genes coding for antigen-presenting molecules, *i.e.* class I and class II, belong to the ancestral MHC [58]. Their genetic variation is functionally important and it was shown to be associated with a variety of diseases. Their complexity and genetic organization require specific methodological approaches, differing between the class I and class II regions. Here, we studied functionally important and usually the most polymorphic class II genes *DRA*, *DRB*, *DQA* and *DQB*, encoding the α and β chains composing functional *DR* and *DQ* antigen-presenting dimers [1].

MHC class II genes are highly variable between different species due to differences in numbers of genes evolved by duplications as well as in the extent of polymorphism between individual genes [59, 60]. Intraspecific variation may be due not only to high numbers of genes and alleles, but also to variation in gene number among individual haplotypes observed in different species, including cattle [61]. It was therefore surprising to observe low variability in the MHC class II exon 2 genomic sequences in the three camelid species. Low numbers of alleles and high numbers of homozygotes in all loci, and many alleles shared across species substantially differ from most other mammalian species with tens or even hundreds of alleles and a high degree of heterozygosity [7]. Based on our observations, we extended the numbers of animals analyzed making sure that the camels studied originated from all major regions in the world (see Additional file 1). Three approaches (Sanger, NGS, cloning) were used to confirm the sequences retrieved and different primer pairs spanning overlapping regions were used to minimize allelic drop-outs. In addition, zoo-primers amplifying canonical MHC class II loci in other species were used. All these approaches produced similar results. The extent of allelic variation identified in groups of animals comparable to studies of other mammalian models seems to be unusually low in this particular family. We only can speculate whether there is another methodological limitation, which avoided us to assess the entire range of existing variation or whether there are reasons related to the biology of this family. Our data did not provide information about the extent of genetic variation in other MHC genes, especially in the class I loci. Lower MHC variation in different species is usually associated with (i) bottlenecks or reduced sizes of specific populations [62], (ii) pathogen-poor environment [63], (iii) particular traits of social interactions such as monogamy combined with small population numbers and low reproduction rate [64], or (iv) limited chance for lateral pathogen transmission [65]. Out of these, the pathogen poor environment is an attractive hypothesis. We observed a lower overall genomic heterozygosity in dromedaries compared to domestic and wild Bactrian camels, which could hint to a generally lower genetic diversity in dromedaries (Fitak et al. in prep). However, for the MHC region, the low level of genetic diversity was observed in all three species.

The whole genome sequences available combined with the results of this study still did not allow us to determine the complete number of different class II loci. In most species, the *DRA* molecule is encoded by a single locus and it is usually monomorphic or with very little variation [66, 67]. Our observation of two SNPs with one non-synonymous substitution is in agreement with this general pattern. All three camel species shared identical alleles, although their distribution differed according to species. Interestingly, the allele DRA*03 prevailing in *C. ferus* (frequency = 0.444) was rarely observed in both domesticated species, *C. bactrianus* (0.05) and *C. dromedarius* (0.067).

The DR*β* chain is usually encoded by multiple highly polymorphic loci [67]. Hundreds of alleles and variation in the numbers of loci were observed in primates [6]. However, in some species like musk-ox and fallow deer, the *DRB* genes were found to be monomorphic [60]. In the three camel species, this was the only locus where species-specific SNPs were observed and it seems to be the least conserved out of the loci studied. This corresponds to high inter-species variability observed in other mammals [68]. The lowest numbers of *DRB* alleles were found in *C. ferus*, which is probably due to the limited number of individuals sampled of this species, originating from a single population and to the fact that wild camels experienced a population reduction of 80 % over the last 100 years [69].

In most mammals, the *DQA* region is typically multi-locus and highly polymorphic [70]. In cattle, variation in the number of *DQA* genes among individual haplotypes were reported [71–73]. Our *in silico* search of the camelid genomes revealed two possible *DQA* loci. However, the lower scoring locus seems to be a non-functional copy, as no other MHC genes could be found within the same scaffold (data not shown). Although several SNPs allowing multiple haplotype combinations were observed in the camel exon 2 *DQA* nucleotide sequences, only three allelic haplotypes were observed, two of them shared by all three camel species although with different frequencies (Table 5). The *DQA* sequences were also highly similar to human sequences (0.843 and 0.780 for nucleotide and peptide sequences, respectively), and in contrast to the *DR* loci, they showed high similarities with their cattle and pig orthologues (Table 7).

The *DQB* genomic region seems to be very complex in camels like in many other mammalian species, with many repetitive sequences of different types [7]. For this reason probably, the camel whole genome sequences published so far do not contain this region. The fact that we were unable to amplify full-length exon 2 sequences by using several pairs of *DQB* zoo-primers (human, cattle, equine) and that even high-coverage whole genome sequencing and/or NGS did not allow resolving the organization of the *DQB* region, is in agreement with this assumption. In this situation, we had to use the zoo-primers GH 28 and 29, shown previously to amplify exon 2 *DQB* sequences from at least two loci of multiple mammalian species [36, 74]. The partial *DQB* exon 2 sequences obtained here contained high numbers of SNPs, with many non-synonymous substitutions. However, they showed the same patterns like the *DQA* locus, with very few individual haplotypes. As the *DQB* sequences obtained here are truncated, some additional variation may exist in these putative loci. A unique feature of the *DQB* exon 2 sequences is the 12 bp insertion not observed in other mammalian species. Based on *in silico* translation, the putative polypeptide may be fully functional.

The study of highly polymorphic immune genes in ancient human (Denisovan) DNA samples revealed that adaptive introgression of archaic alleles has significantly shaped the modern human immune system [75]. Analyses of MHC genes from ancient domestic animal specimens thus can help to elucidate the historical events occurring during the domestication process. Most ancient DNA studies in domestic animals relied on mtDNA due to general difficulties associated with the recovery of nuclear DNA (nuDNA) from archaeozoological materials. Despite their importance, there have been no studies of ancient immune genes and their role in domestication and adaptation in livestock to date. This dataset represents the first MHC sequences retrieved from ancient camel specimens from hot and arid environments, which are notoriously the most unfavorable for the survival of DNA [76]. Although fragmentary at this stage, the data showed that some *DRA* haplotypes have been maintained over time. The three extra C/T substitutions observed in AQ30 still need to be validated as true polymorphisms in additional (historical) samples, rather than representing ancient DNA damage commonly observed as C to T (complementary G to A) change resulting from post-mortem cytosine deamination [77].

Previously, Antczak [23] reported on the existence of polymorphic MHC class I and class II genes and on MHC-linked microsatellite repeats located in the Bactrian camel genome. A whole genome cytogenetic map including data on the MHC region is available for New World camelids [57]. However, no specific sequences were available for a comparison for the purposes of this study.

## Conclusion

In summary, this study provided the first evidence that camels possess an MHC comparable to other mammalian species in terms of its genomic localization, organization and sequence similarity. This is the first complex report on the order of the three MHC major sub-regions in Old World camels, based on physical mapping. We described ancient variation at the *DRA* locus, monomorphic in most species. The extent of molecular diversity of MHC class II genes seems to be substantially lower in Old World camels than in other mammalian species. The major part of the diversity could reside in the complex *DQB* region, which was difficult to resolve and remained unannotated in the whole genome sequences.

### Availability of supporting data

All alignments and resulting phylogenetic trees are available at the TreeBASE (http://treebase.org/treebase-web/home.html) under DOI http://purl.org/phylo/treebase/phylows/study/TB2:S18850.

All sequences were submitted to GenBank (http://www.ncbi.nlm.nih.gov/genbank/) and are available under accession numbers KT936396-KT936421.

## Additional files

Additional file 1: List of animals included in the study. (PDF 220 kb) Additional file 2: Chromatograms of selected DRA alleles. (PDF 218 kb) Additional file 3: Chromatograms of selected DRB alleles. Rest of the alleles was inferred. (PDF 332 kb) Additional file 4: Chromatograms of selected DQA alleles. (PDF 156 kb) Additional file 5: Chromatograms of selected DQB sequences. (PDF 77 kb)
