# Supplementary material for: The major histocompatibility complex in Old World camelids and low polymorphism of its class II genes
Source: BMC Genomics. 2016 Mar 1;17:167. doi: 10.1186/s12864-016-2500-1 (PMC4774177; doi:10.1186/s12864-016-2500-1)
Supplement: Additional file 1: — List of animals included in the study. (PDF 220 kb) [file 12864_2016_2500_MOESM1_ESM.pdf]

Additional file 1: List of animals included in the study.

ID	Species	Location	DQA	DRA	DQB	DRB	DYA
WC 4	Camelus ferus	Mongolia		x		x	
WC 21	Camelus ferus	Mongolia		x			
WC 111	Camelus ferus	Mongolia	x				
WC 116	Camelus ferus	Mongolia		x		x	
WC 134	Camelus ferus	Mongolia		x			
WC 132B	Camelus ferus	Mongolia		x		x	
WC 150	Camelus ferus	Mongolia		x			
WC 157A	Camelus ferus	Mongolia		x		x	
WC20	Camelus ferus	Mongolia	x	x			
WC 1	Camelus ferus	Mongolia		x	x	x	
WC 2	Camelus ferus	Mongolia		x	x	x	
WC 3	Camelus ferus	Mongolia	x	x	x	x	
WC 5	Camelus ferus	Mongolia	x	x			
WC 6	Camelus ferus	Mongolia	x	x	x	x	
WC 11	Camelus ferus	Mongolia	x	x	x	x	
WC 16	Camelus ferus	Mongolia	x	x			
WC 10	Camelus ferus	Mongolia		x			
WC 19	Camelus ferus	Mongolia	x	x		x	
WC 191	Camelus ferus	China				x	
cWC 5	Camelus ferus	China		x			
Total			8	18	5	11	0
ID	Species	Location	DQA	DRA	DQB	DRB	DYA
Drom 1	Camelus dromedarius	Kenya				x	
Drom 2	Camelus dromedarius	Kenya				x	
Drom 5	Camelus dromedarius	Kenya				x	
Drom 8	Camelus dromedarius	Kenya				x	
Drom 10	Camelus dromedarius	Kenya				x	
Drom 11	Camelus dromedarius	Kenya				x	
Drom 13	Camelus dromedarius	Kenya				x	
Drom 40	Camelus dromedarius	Yemen				x	
Drom 42	Camelus dromedarius	Yemen	x				
Drom 43	Camelus dromedarius	Yemen	x				
Drom 46	Camelus dromedarius	Yemen				x	
Drom 54	Camelus dromedarius	Sudan	x				
Drom 56	Camelus dromedarius	Sudan	x				
Drom 120	Camelus dromedarius	Libya				x	
Drom 139	Camelus dromedarius	Libya				x	
Drom 144	Camelus dromedarius	Libya				x	
Drom 494	Camelus dromedarius	Algeria				x	
Drom 602	Camelus dromedarius	Niger	x			x	
Drom 607	Camelus dromedarius	Niger				x	
Drom 624	Camelus dromedarius	Niger				x	
Drom 758	Camelus dromedarius	Sudan	x				

Drom 150	Camelus dromedarius	Australia		x		x	
Drom 151	Camelus dromedarius	Australia		x		x	
Drom 152	Camelus dromedarius	Australia				x	
Drom 153	Camelus dromedarius	Australia				x	
Drom 154	Camelus dromedarius	Australia		x		x	
Drom 155	Camelus dromedarius	Australia					
Drom 156	Camelus dromedarius	Australia		x		x	
Drom 157	Camelus dromedarius	Australia					
Drom 158	Camelus dromedarius	Australia		x			
Drom 159	Camelus dromedarius	Australia		x		x	
Drom 160	Camelus dromedarius	Australia		x			
Drom 161	Camelus dromedarius	Australia					
Drom 162	Camelus dromedarius	Australia				x	
Drom 163	Camelus dromedarius	Australia				x	
J12	Camelus dromedarius	Jordan	x	x		x	x
J13	Camelus dromedarius	Jordan	x	x	x	x	x
J15	Camelus dromedarius	Jordan		x		x	x
J18	Camelus dromedarius	Jordan		x		x	x
J33	Camelus dromedarius	Jordan	x	x	x	x	x
J41	Camelus dromedarius	Jordan		x		x	
J43	Camelus dromedarius	Jordan		x	x	x	x
J45	Camelus dromedarius	Jordan		x		x	x
J61	Camelus dromedarius	Jordan		x		x	x
J65	Camelus dromedarius	Jordan		x	x	x	
J66	Camelus dromedarius	Jordan		x			
J103	Camelus dromedarius	Jordan	x	x	x	x	
J104	Camelus dromedarius	Jordan	x	x	x	x	
J105	Camelus dromedarius	Jordan	x	x	x	x	
J106	Camelus dromedarius	Jordan		x		x	
J108	Camelus dromedarius	Jordan		x		x	
J109	Camelus dromedarius	Jordan	x	x		x	
J116	Camelus dromedarius	Jordan	x	x		x	
J125	Camelus dromedarius	Jordan	x				x
J131	Camelus dromedarius	Jordan	x	x		x	
J132	Camelus dromedarius	Jordan	x	x		x	
J133	Camelus dromedarius	Jordan		x		x	
J135	Camelus dromedarius	Jordan					x
J158	Camelus dromedarius	Jordan	x	x		x	x
J163	Camelus dromedarius	Jordan		x		x	
J165	Camelus dromedarius	Jordan		x		x	
J175	Camelus dromedarius	Jordan		x		x	
J179	Camelus dromedarius	Jordan		x		x	
J180	Camelus dromedarius	Jordan		x		x	x
J185	Camelus dromedarius	Jordan	x	x		x	x
J188	Camelus dromedarius	Jordan		x		x	
J195	Camelus dromedarius	Jordan		x		x	

Total			19	37	7	54	13
ID	Species	Location	DQA	DRA	DQB	DRB	DYA
DC 275	Camelus bactrianus	China	x				
M184	Camelus bactrianus	Mongolia		x		x	x
M185	Camelus bactrianus	Mongolia	x			x	
M186	Camelus bactrianus	Mongolia	x	x		x	
M187	Camelus bactrianus	Mongolia				x	x
M188	Camelus bactrianus	Mongolia	x	x		x	
M189	Camelus bactrianus	Mongolia	x			x	
M190	Camelus bactrianus	Mongolia	x	x		x	x
M191	Camelus bactrianus	Mongolia	x			x	
M192	Camelus bactrianus	Mongolia	x	x		x	
M193	Camelus bactrianus	Mongolia	x			x	x
M219	Camelus bactrianus	Mongolia				x	
M220	Camelus bactrianus	Mongolia	x	x		x	x
M222	Camelus bactrianus	Mongolia	x	x		x	
M223	Camelus bactrianus	Mongolia	x			x	
M251	Camelus bactrianus	Mongolia	x	x			
M252	Camelus bactrianus	Mongolia	x	x			x
M253	Camelus bactrianus	Mongolia	x				
M254	Camelus bactrianus	Mongolia	x	x			
M255	Camelus bactrianus	Mongolia	x				
M256	Camelus bactrianus	Mongolia	x	x			
M258	Camelus bactrianus	Mongolia	x				x
M259	Camelus bactrianus	Mongolia	x				
M260	Camelus bactrianus	Mongolia		x			
M174	Camelus bactrianus	Mongolia	x	x	x		
M175	Camelus bactrianus	Mongolia	x		x	x	
M176	Camelus bactrianus	Mongolia	x	x	x	x	
M177	Camelus bactrianus	Mongolia	x		x	x	
M34	Camelus bactrianus	Mongolia	x		x	x	
M35	Camelus bactrianus	Mongolia	x	x	x	x	
M36	Camelus bactrianus	Mongolia	x		x	x	
M53	Camelus bactrianus	Mongolia	x	x	x	x	x
M55	Camelus bactrianus	Mongolia	x			x	
M56	Camelus bactrianus	Mongolia	x	x	x	x	
M149	Camelus bactrianus	Mongolia				x	
M150	Camelus bactrianus	Mongolia	x	x	x	x	
M151	Camelus bactrianus	Mongolia	x			x	x
M153	Camelus bactrianus	Mongolia	x		x	x	
M154	Camelus bactrianus	Mongolia	x	x	x	x	
M152	Camelus bactrianus	Mongolia	x	x	x	x	
M155	Camelus bactrianus	Mongolia				x	
M156	Camelus bactrianus	Mongolia		x		x	
M157	Camelus bactrianus	Mongolia	x		x	x	x

M158	Camelus bactrianus	Mongolia	x	x		x	
M159	Camelus bactrianus	Mongolia	x			x	
M160	Camelus bactrianus	Mongolia		x		x	
M163	Camelus bactrianus	Mongolia	x				
M164	Camelus bactrianus	Mongolia	x	x		x	
M165	Camelus bactrianus	Mongolia				x	
M166	Camelus bactrianus	Mongolia	x	x		x	
M167	Camelus bactrianus	Mongolia	x			x	
M168	Camelus bactrianus	Mongolia	x	x			
M169	Camelus bactrianus	Mongolia	x				
M170	Camelus bactrianus	Mongolia	x	x			
M172	Camelus bactrianus	Mongolia	x	x	x		
M178	Camelus bactrianus	Mongolia	x	x	x	x	
M179	Camelus bactrianus	Mongolia			x	x	x
M180	Camelus bactrianus	Mongolia		x	x	x	
Total			47	30	18	42	11
