# Supplementary material for: The major histocompatibility complex in Old World camelids and low polymorphism of its class II genes
Source: BMC Genomics. 2016 Mar 1;17:167. doi: 10.1186/s12864-016-2500-1 (PMC4774177; doi:10.1186/s12864-016-2500-1)

DRA*01

File: 166CBAB003-17.ab1 *Run Ended: 2014/8/28 19:46:20* *Signal G:3516 A:3943 C:5278 T:4641*

Sample: 166CBAB003-17_premix *Lane: 63* *Base spacing: 14.86778* *1086 bases in 13184 scans*

MACROGEN
Advancing through Genomics

10 20 30 40 50 60 70 80 90 100 110 120
GTATTCTGGACAGAGAGACGTTTCTCTTTTAGGAGATGCTGCTGGATTTCTAAAGAGGAAATCATTTCTTGGCCAGTAACATGGAGCCAAACAAAATCTTCATGAGACTCTGGGTGTTTCA

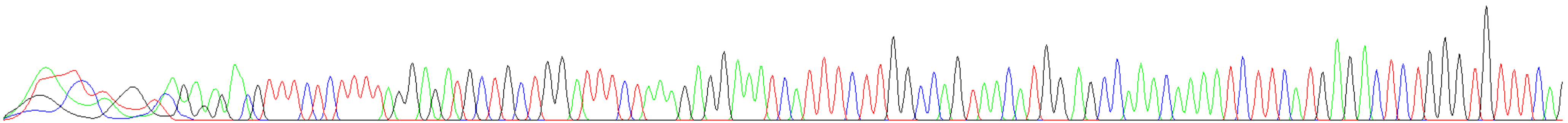

130 140 150 160 170 180 190 200 210 220 230 240
GCTCTTGTGTCCACACTCTCTGCGTCACCCCTTCATTCCTCCACTTCCACCTCTGAGCAACACGAACGTCCTTCACCCGGCCTCCTCCTCCTGCACAGCCCTGCTCTCCTTCACTCCTCC

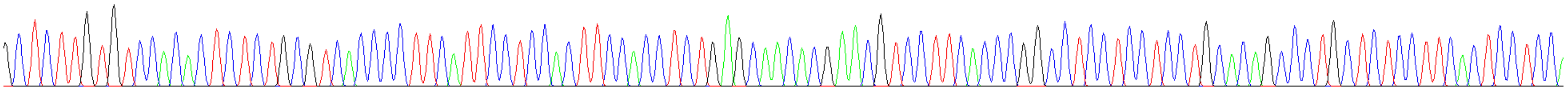

250 260 270 280 290 300 310 320 330 340 350 360
ACTCCACTTGACCTCTCTTCCCACCACTCCCGCCCACGTGTTTGTGCCTCACATCCTGGGTCCTCCCTCTCTCTCCTGGTTCCTACCCTAATTCCCTTTCTTGTCTTTTCAGAGGAACACGT

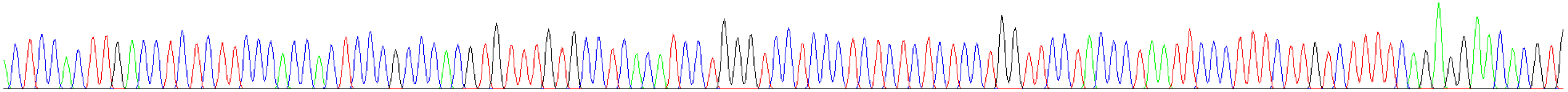

370 380 390 400 410 420 430 440 450 460 470 480 490
GATCATCCAGGCTGAGTTCTATCTGAACCCTGACAAGTCAGGCGAGTATATGTTTGACTTTGACGGTGATGAGATTTTCCACGTGGATCTGGAAAAGAAGGAGACGGTCTGGCGGCTTGAAGAAT

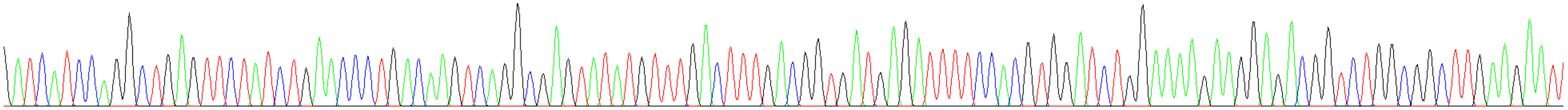

500 510 520 530 540 550 560 570 580 590 600 610
TTGGACGTTTTGCCAGCTTTGAGGCTCAGGGTGCATTGGCCAATATAGCTGTGGACAAAGCCAACCTGGACATCATGATAAAGCGCTCCAACCACACCCCGAACACCAATGGTACCTCTCTCTGCT

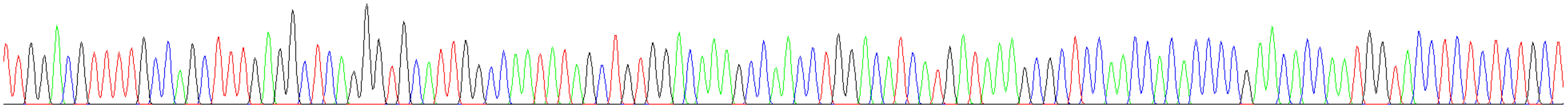

File: 166CBAB003-17.ab1 *Run Ended: 2014/8/28 19:46:20* *Signal G:3516 A:3943 C:5278 T:4641*

Sample: 166CBAB003-17_premix *Lane: 63* *Base spacing: 14.86778* *1086 bases in 13184 scans*

620 630 640 650 660 670 680 690 700 710 720 730 740

GCCCTCCTAGACGTGGGAATTGTAGCTTTAGATAGATGCTCAGCTCTCTCTGTTGTGTTATCGTGACTGACTTGCCCTTCCAGGGGCCTGACCTTGCCATAAGCAAACCCAAAATTCTCACATTA

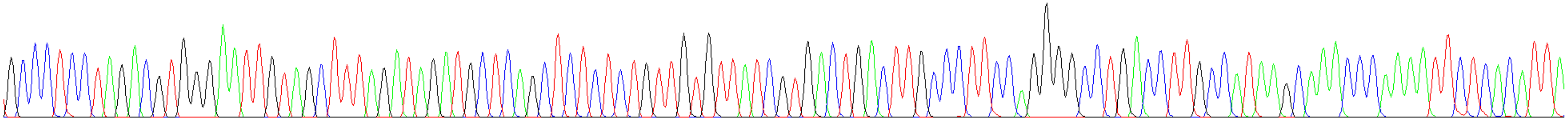

750 760 770 780 790 800 810 820 830 840 850 860

CCAACCCAAGAACTTCATGAGTTTGTTCCTTTCTGGGTGTGCTCACATCTTGTCTTTGCCACCCATGGTCTCTCATAAAGTCTTTGCCTGAATCCATGCTTCCATGCTGGGGGAGCCAAGAAT

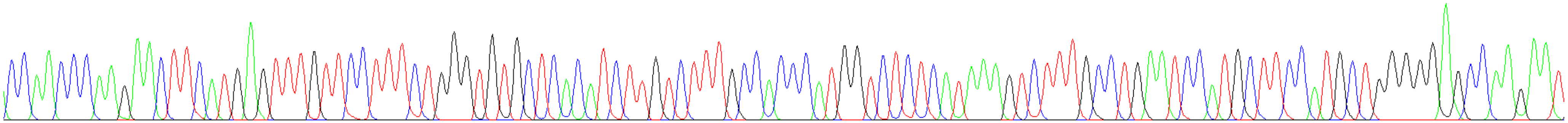

870 880 890 900 910 920 930 940 950 960 970 980

GCGGTCCTTGGAGACCTTGAACCTCAGCATCGGTCGCTGTTCATGGGGAGGGTGTCAGAGCTGGGTATCTGAGACCATGGCGCAGATTCTTAGGACACAGCTTTTTTTTTTATACACACACAAAC

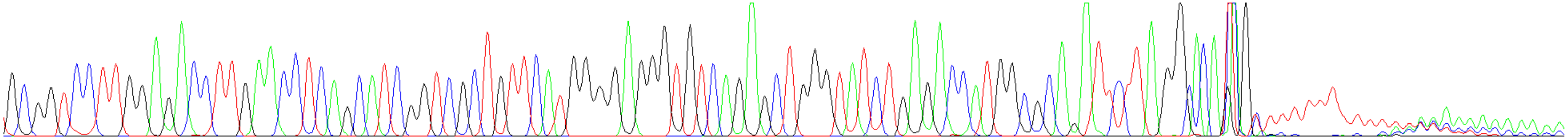

990 1000 1010 1020 1030 1040 1050 1060 1070 1080

AAAAAAATAAAAAAAAAACCCACTCCTCCCCCTTCCGCTTCGTCCCCCGCCTCCCCCCCTTTCCTTTTCCCTTCTTTCTTTCCCCGCCTCTCCTCTTC

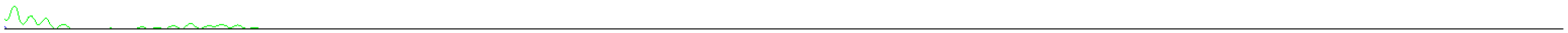

DRA*02

File: 13EFBAA003-47.ab1   *Run Ended: 2013/2/15 8:3:15*   *Signal G:3412 A:4210 C:3345 T:3010*

Sample: 13EFBAA003-47_premix   *Lane: 13*   *Base spacing: 15.086332*   *1437 bases in 16303 scans*

MACROGEN Advancing through Genomics

10 20 30 40 50 60 70 80 90 100 110 120

ACCAGTTCAGGNCCCTGGTAGGGCAGTCAGTCACGATAACACAACAGAGAGAGCTGAGCATCTATCTAAAGCTACAATTCCCACGTCTAGGAGGGCAGCAGAGAGAGGTACCATTGGTGTTCGGGGTG

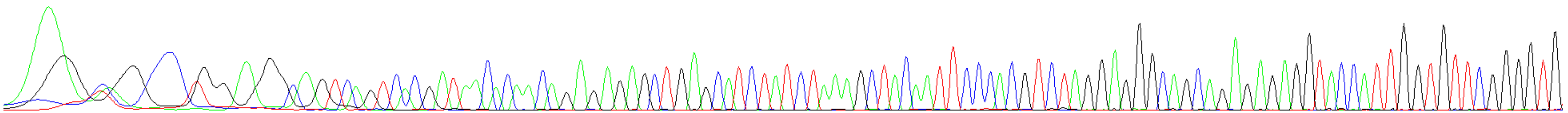

130 140 150 160 170 180 190 200 210 220 230 240 250

TGGTTGGAGCGCTTTATCATGATGTCCAGGTTGGCTTTGTCCACAGCTATATTGGCCAATGCACCCTGAGCCTCAAAGCTGGCAAACCGTCCAAATTCTTCAAGCCGCCAGACCGTCTCCTTCTTT

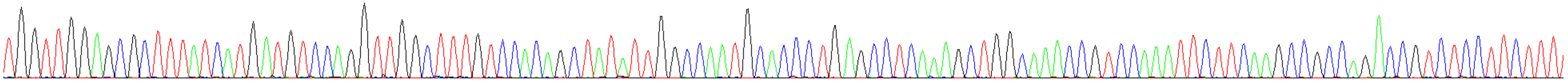

260 270 280 290 300 310 320 330 340 350 360 370 380

TCCAGATCCACGTGGAAAATCTCATCACCGTCAAAGTCAAACATAAACTCGCCTGACTTGTCAGGGTTCAGATAGAACTCAGCCTGGATGATCACGTGTTCCTCTGAAAAGACAAGAAAGGGAATT

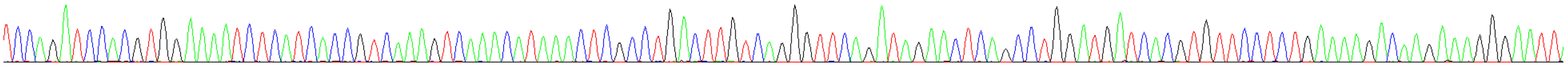

390 400 410 420 430 440 450 460 470 480 490 500

AGGGTAGGAACCAGGAGAGAGAGAGGGAGGACCCAGGATGTGAGGCACAAACACGTGGGCAGGAGTGGTGGGAAGAGAGGCCAAGTGGAGTGGAGGAGTGAAGGAGAGCAGGGCTGTGCAGGAG

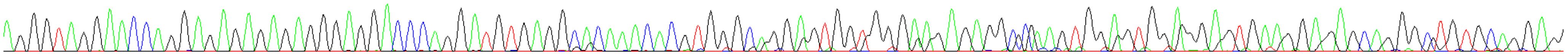

510 520 530 540 550 560 570 580 590 600 610 620 630

GAGGAGGCCGGGTGAAGGACGTTCGTTGCCTCTCAAGGGAAGANAANNNNNNNNNNNNNNNNNNNNNNNNNNNNNNNNNNNNNNNNNNNNNNNNNNNNNNNNNNNNNNNNNNNNNNNNNNNNNNNNNNNNNN

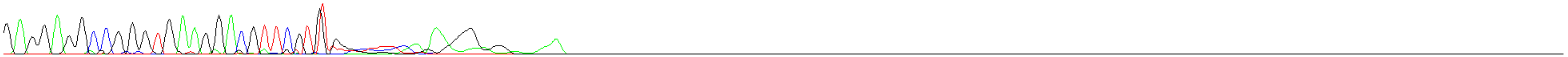

DRA*03

File: 166CBAB000-9.ab1   Run Ended: 2014/7/22 18:33:43   Signal G:5390 A:5788 C:6118 T:4400

Sample: 166CBAB000-9_premix   Lane: 62   Base spacing: 14.735186   1195 bases in 16299 scans

MACROGEN
Advancing through Genomics

10 20 30 40 50 60 70 80 90 100 110 120

C GGCA AAG A CAC A CTAA AG A C CAA T CA TAAA GT T CT T G G GT T G GTAAT GT GAGAATTTT GGGTTTGCTTATGGCAAGGTCAGGCCCCTGGAAGGGCAAGTCAGTCACGATAACACAACAG

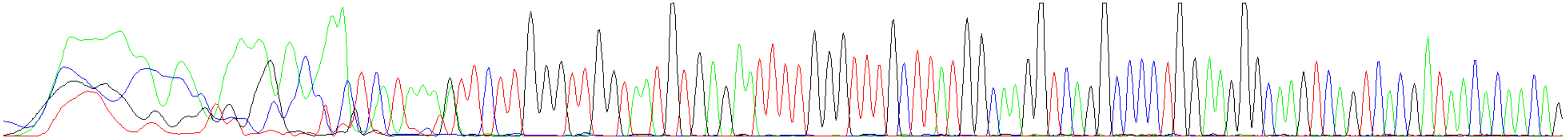

130 140 150 160 170 180 190 200 210 220 230 240

AGAGAGCTGAGCATCTATCTAAAGCTACAATTCCCACGTCTAGGAGGGCAGCAGAGAGAGGTACCATTGGTGTTCGGGGTGTGGTTGGAGCGCTTTATCATGATGTCCAGGTTGGCTTTGTCCAC

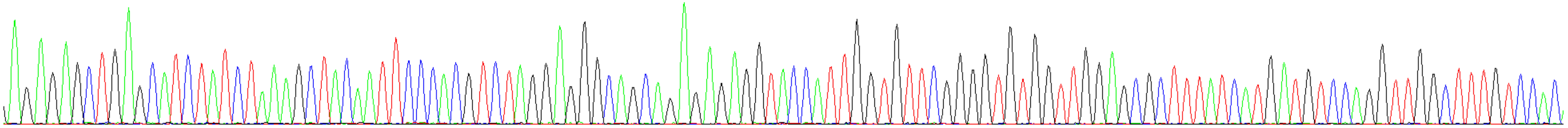

250 260 270 280 290 300 310 320 330 340 350 360 370

AGCTATATTGGCCAATGCACCCTGAGCCTCAAAGCTGGCAAAACGTCCAAATTCTTCAAGCCGCCAGACCGTCTCCTTCTTTTCCAGATCCACGTGGAAAATCTCATCACCGTCAAAGTCAAACAT

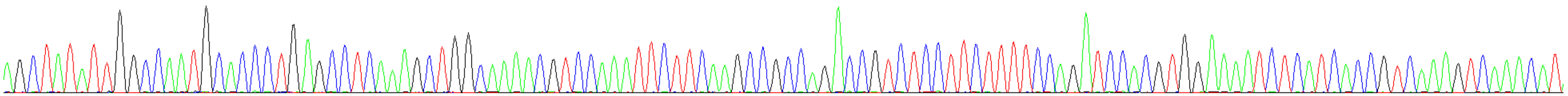

380 390 400 410 420 430 440 450 460 470 480 490

AAACTCGCCTGACTTGTCAGGGTTCAGATAGAACTCAGCCTGGATGATCACGTGTTCCTCTGAAAAGACAAGAAAGGGAATTAGGGTAGGAACCAGGAGAGAGAGAGGGAGGACCCAGGATGTGA

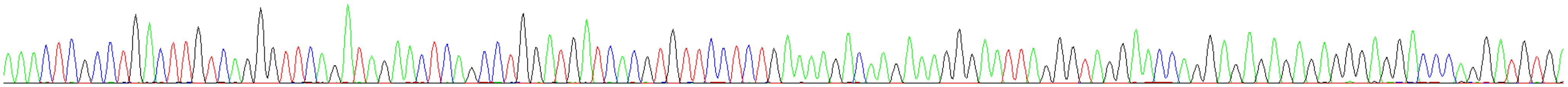

500 510 520 530 540 550 560 570 580 590 600 610 620

GGCACAAACACGTGGGCAGGAGTGGTGGGAAGAGAGGCCAAGTGGAGTGGAGGAGTGAAGGAGAGCAGGGCTGTGCAGGAGGAGGAGGCCGGGTGAAGGACGTTCGTGTTGCTCAGAGGTGGAAG

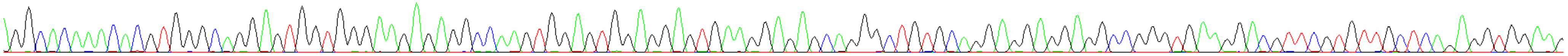

Supplement: Additional file 2: — Chromatograms of selected DRA alleles. (PDF 218 kb) [file 12864_2016_2500_MOESM2_ESM.pdf]
