# Supplementary material for: The major histocompatibility complex in Old World camelids and low polymorphism of its class II genes
Source: BMC Genomics. 2016 Mar 1;17:167. doi: 10.1186/s12864-016-2500-1 (PMC4774177; doi:10.1186/s12864-016-2500-1)

DRB*01

File: 166CBAB000-53.ab1   Run Ended: 2014/7/22 20:31:24   Signal G:1180 A:1058 C:929 T:772

Sample: 166CBAB000-53_premix   Lane: 5   Base spacing: 14.749477   1364 bases in 16303 scans

Advancing through Genomics

10 20 30 40 50 60 70 80 90 100 110 120

CCC G CTTGG GGT TGG G CTG G A GCANAGTGACTGCATCCCAAATGGCTTANGATCAGAGGAGTCTCCTCCCCCCAGGACGGCCTTCCTCCACCACTGGCAGGTGAAAGATGGACCCCTGGGGTGC

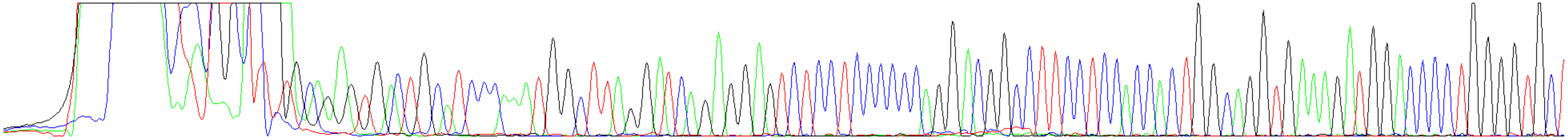

130 140 150 160 170 180 190 200 210 220 230 240 250

TTGTGCTGGTGCTTAGGGCCCCAGCGGAGCAGTGGGGGTCCTAGTGCCCCCAGCAGTGCGTGAGCACTCTGCACCGGTGGGCAGCACTGTCAGTCTTCTCCTACAAGCCCACCCAGTGACCAGATC

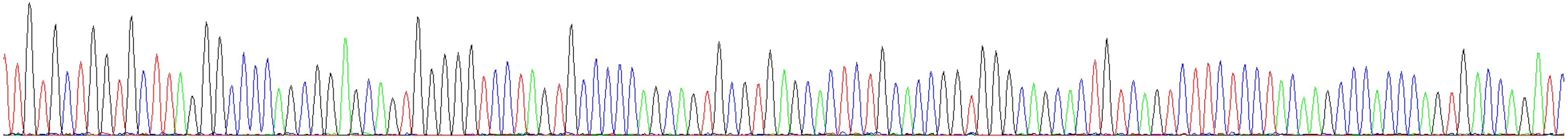

260 270 280 290 300 310 320 330 340 350 360 370

TTTTATGTCCTCACAGCAACTTTCATGCATCAGTTTAAGGGAGAGTGTCGTTTCTTCAACGGCTTGGAGCGGGTGCAGTTCTTGGCCAGATACATCTATAATACAGAGGAGGATGTGTACTTCGA

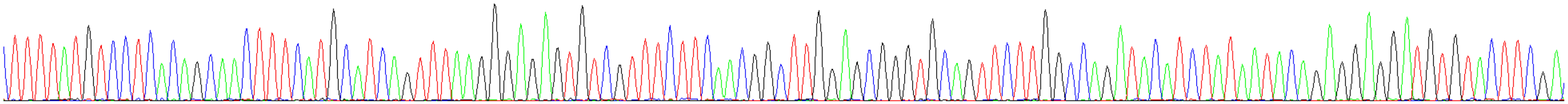

380 390 400 410 420 430 440 450 460 470 480 490 500

CAGCGACGTGGGGGAGTTCAGGGCGGTGACCGAGCTGGGGCAGCCAGACGCCAAGTACTGGAACCAGCAGAAGGACTTCATGGAGCAGATGCGGGCCAAGGTGGACACGCTGTGCAGATCAAACT

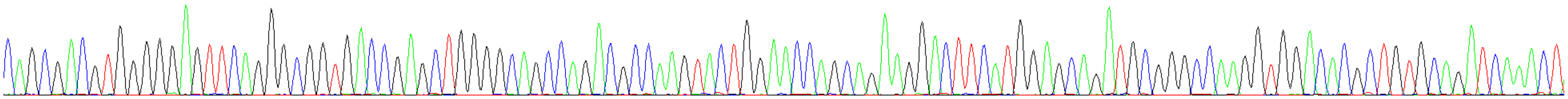

510 520 530 540 550 560 570 580 590 600 610 620

ACTGGGGAATAGGCAGCTTCATGGTGCAGCGGCGAGGTGAGCGGGCAGGCGGGTGGCCGGGGAGAGGTGGTGTGTGTGTGGGGGGTGTGTCTGTGTGTGGGAGAGAGAGGGAGAGAGACAGAG

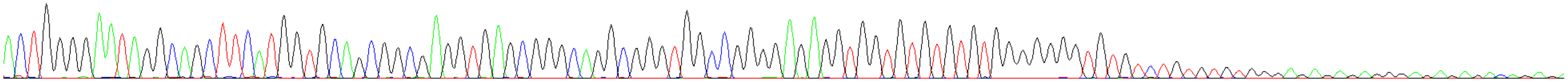

DRB*02/Caba-DRB*03

File: 166CBAB000-64.ab1   *Run Ended: 2014/7/22 20:31:24*   *Signal G:2153 A:1400 C:1413 T:947*

Sample: 166CBAB000-64_premix   *Lane: 31*   *Base spacing: 14.6514015*   *1426 bases in 16302 scans*

MACROGEN Advancing through Genomics

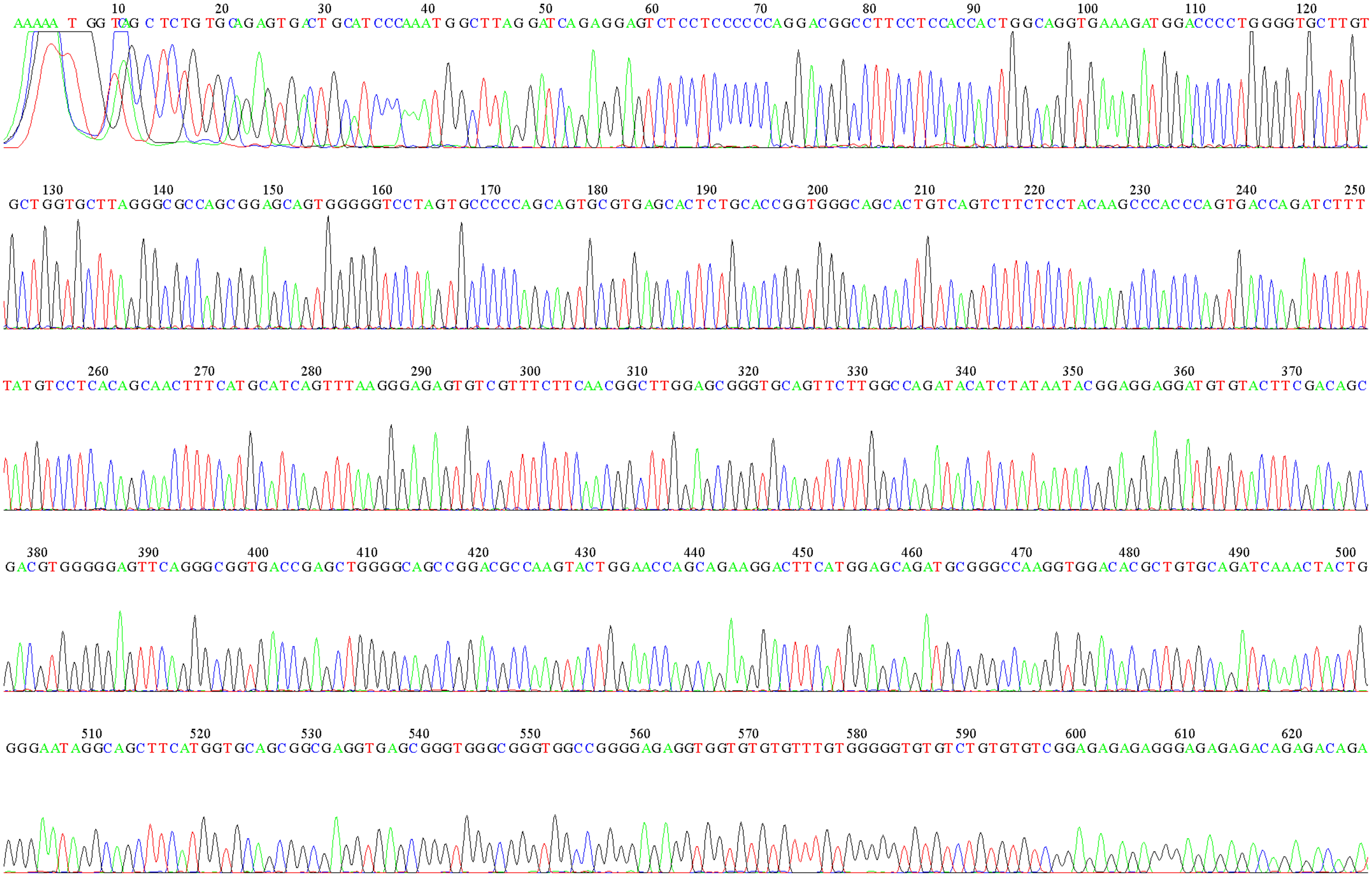

Caba-DRB*01/02 (heterozygote)

File: 13EFBAA009-43.ab1   Run Ended: 2013/8/7 18:13:56   Signal G:8601 A:9343 C:11148 T:8258

Sample: 13EFBAA009-43_premix   Lane: 42   Base spacing: 14.962415   1360 bases in 16303 scans   Page 1 of 2

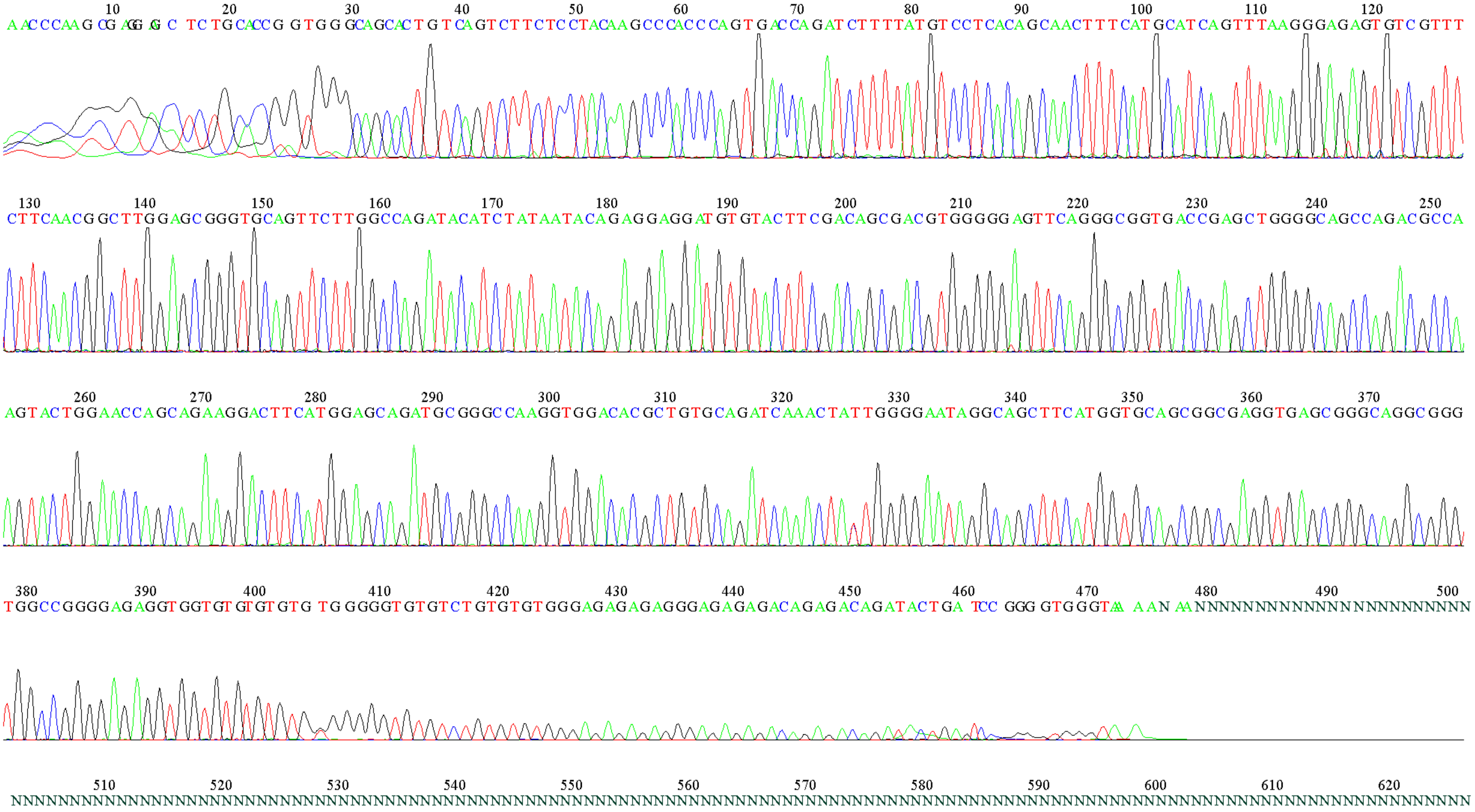

Caba-DRB*03/04 (heterozygote)

File: 13EFBAA010-39.ab1 *Run Ended: 2013/8/16 3:45:39* *Signal G:5496 A:5846 C:7457 T:5229*

Sample: 13EFBAA010-39_premix *Lane: 54* *Base spacing: 14.362148* *559 bases in 6611 scans*

10 20 30 40 50 60 70 80 90 100 110 120
GGAGCCAGCGAGACCTGGGTGGGTGTGGGCAGCACTGTCAGTCTTCTCCTACAAGCCCACCCAGTGACCAGATCTTTTATGTCCTCACAGCAACTTTCATGCATCAGTTTAAGGGAGAGTGTCGTT

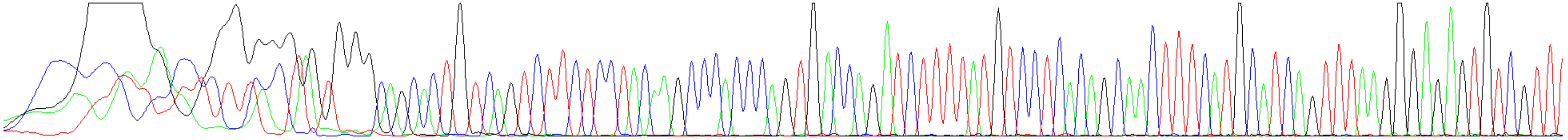

130 140 150 160 170 180 190 200 210 220 230 240 250
TCTTCAACGGCTTGGAGCGGGTGCAGTTCTTGGCCAGATACATCTATAATACGGAGGAGGATGTGTACTTCGACAGCGACGTGGGGGAGTTCAGGGCGGTGACCGAGCTGGGGCAGCCGGACGCCA

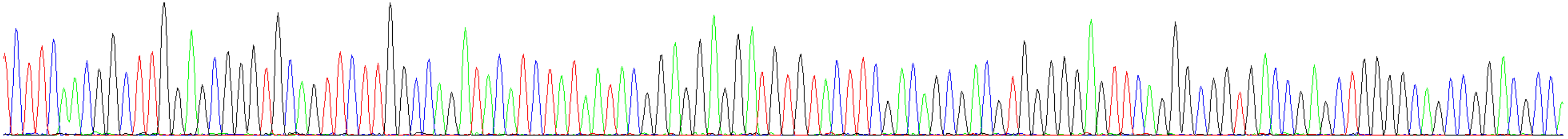

260 270 280 290 300 310 320 330 340 350 360 370
AGTACTGGAACCAGCAGAAGGACTTCATGGAGCAGATGCGGGCCAAGGTGGACACGCTGTGCAGATCAAACTACTGGGGAATAGGCAGCTTCATGGTGCAGCGGCGAGGTGAGCGGGTGGGCGGG

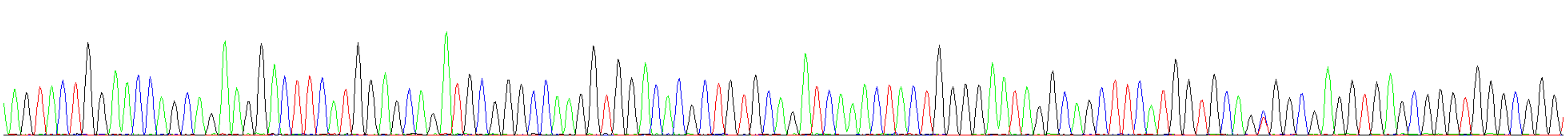

380 390 400 410 420 430 440 450 460 470 480 490 500
TGGCCGGGGAGAGGTGGTGTGTGTGTGTGGGGGTGTGTCTGTGTGTCGGAGAGAGAGGGAGAGAGACAGAGACAGATACTGAGTCCGGGGTGGGTAAAAANNNNNNNNNNNNNNNNNNNNNNNNNNN

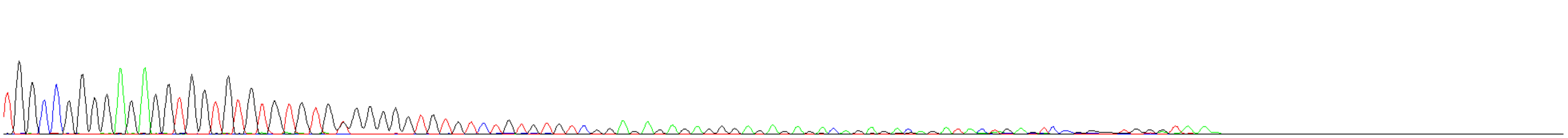

510 520 530 540 550
NNNNNNNNNNNNNNNNNNNNNNNNNNNNNNNNNNNNNNNNNNNNNNNANNNNNNNN

Cadr-DRB*03

File: 16BABAA005-27.ab1 *Run Ended: 2014/12/19 8:27:7* *Signal G:1607 A:1881 C:1842 T:1667*

Sample: 16BABAA005-27_premix *Lane: 47* *Base spacing: 14.49237* *886 bases in 10707 scans*

MACROGEN
Advancing through Genomics

C GCCTGGC GGCTATGTTTGGGCTGAGAGGAGGCAGCAGCGTCTTTCTGGTGGAGGCATTCAGGAGGAGGTGGTGATGGGGCAAATCCTGGTCCAGCTCTGTGCAGAGTGACTGCATCCCAAAT

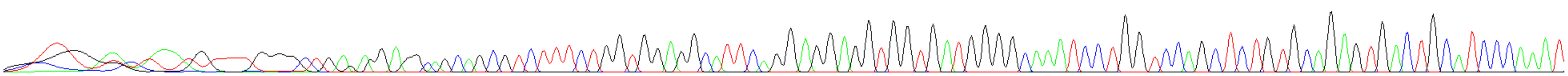

GGCTTAGGATCAGAGGAGTCTCCTCCCCCCAGGACGGCCTTCCTCCACCACTGGCAGGTGAAAGATGGACCCCTGGGGTGCTTGTGCTGGTGCTTAGGGCCCCAGCGGAGCAGTGGGGGTCC

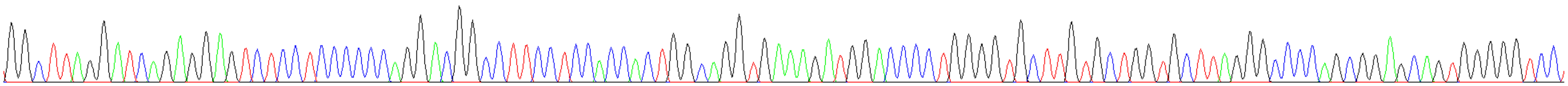

TAGTGCCCCCAGCAGTGCGTGAGCACTCTGCACCGGTGGGCAGCACTGTCAGTCTTCTCCTACAAGCCCACCCAGTGACCAGATCTTTTATGTCCTCACAGCAACTTTCATGCATCAGTTTAAGGG

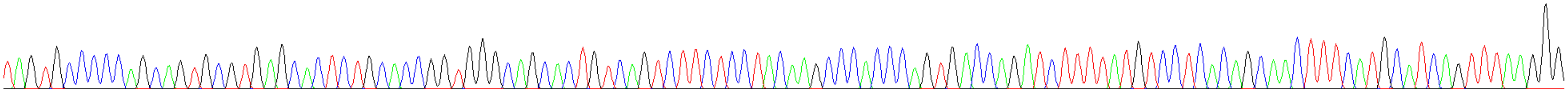

AGAGTGTCGTTTCTTCAACGGCTTGGAGCGGGTGCAGTTCTTGGCCAGATACATCTATAATACAGAGGAGGATGTGTACTTCGACAGCGACGTGGGGGAGTTCAGGGCGGTGACCGAGCTGGGGC

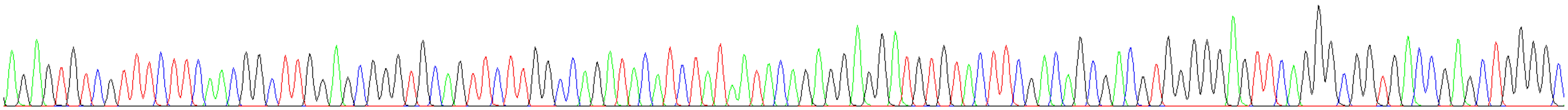

AGCCAGACGCCAAGTACTGGAACCAGCAGAAGGACTTCATGGAGCAGATGCGGGCCAAGGTGGACACGCTGTGCAGATCAAACTATTGGGGAATAGGCAGCTTCATGGTGCAGCGGCGAGGTGAG

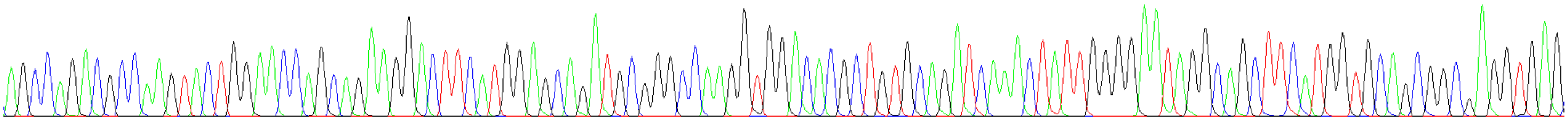

File: 16BABAA005-27.ab1 *Run Ended: 2014/12/19 8:27:7* *Signal G:1607 A:1881 C:1842 T:1667*

Sample: 16BABAA005-27_premix *Lane: 47* *Base spacing: 14.49237* *886 bases in 10707 scans*

630 640 650 660 670 680 690 700 710 720 730 740

CGGGCAGGCGGGTGGCCGGGGAGAGGTGGTGTGTGTGTGTGGGGGGTGTGTCTGTGTGTGGGAAAGAGAGGGAGAGAGACAGAGACAGATACTGAGTCCGGGTGGGTGGGATGCACTGTGAGCCA

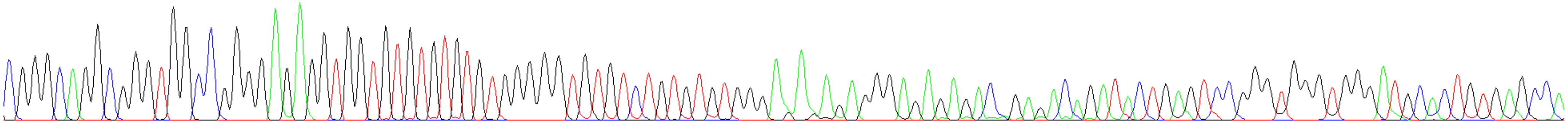

750 760 770 780 790 800 810 820 830 840 850 860

GTATAATTTAGGAAGGAGCTGAAAAAGGTGTTGTTGGGGAGACAGTGCGTGACTTCAGCAGTGACTGTGAGAAGGGAGAGAGGATGTGGTTGGGGGAATGGGTCTGGGCTGAGAGAAAAAAAA

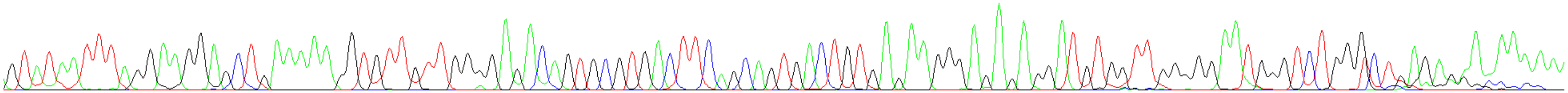

870 880

AAAAAAAAGCGAAAAGA

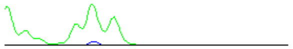

Supplement: Additional file 3: — Chromatograms of selected DRB alleles. Rest of the alleles was inferred. (PDF 332 kb) [file 12864_2016_2500_MOESM3_ESM.pdf]
