# Supplementary material for: The major histocompatibility complex in Old World camelids and low polymorphism of its class II genes
Source: BMC Genomics. 2016 Mar 1;17:167. doi: 10.1186/s12864-016-2500-1 (PMC4774177; doi:10.1186/s12864-016-2500-1)
Supplement: Additional file 4: — Chromatograms of selected DQA alleles. (PDF 156 kb) [file 12864_2016_2500_MOESM4_ESM.pdf]

# DQA*01

*File: 13EFBAA005-21.ab1* *Run Ended: 2013/3/21 6:29:5* *Signal G:5407 A:4445 C:4313 T:4954*

*Sample: 13EFBAA005-21_premix* *Lane: 40* *Base spacing: 14.377768* *1451 bases in 16299 scans* *Page 1 of 2*

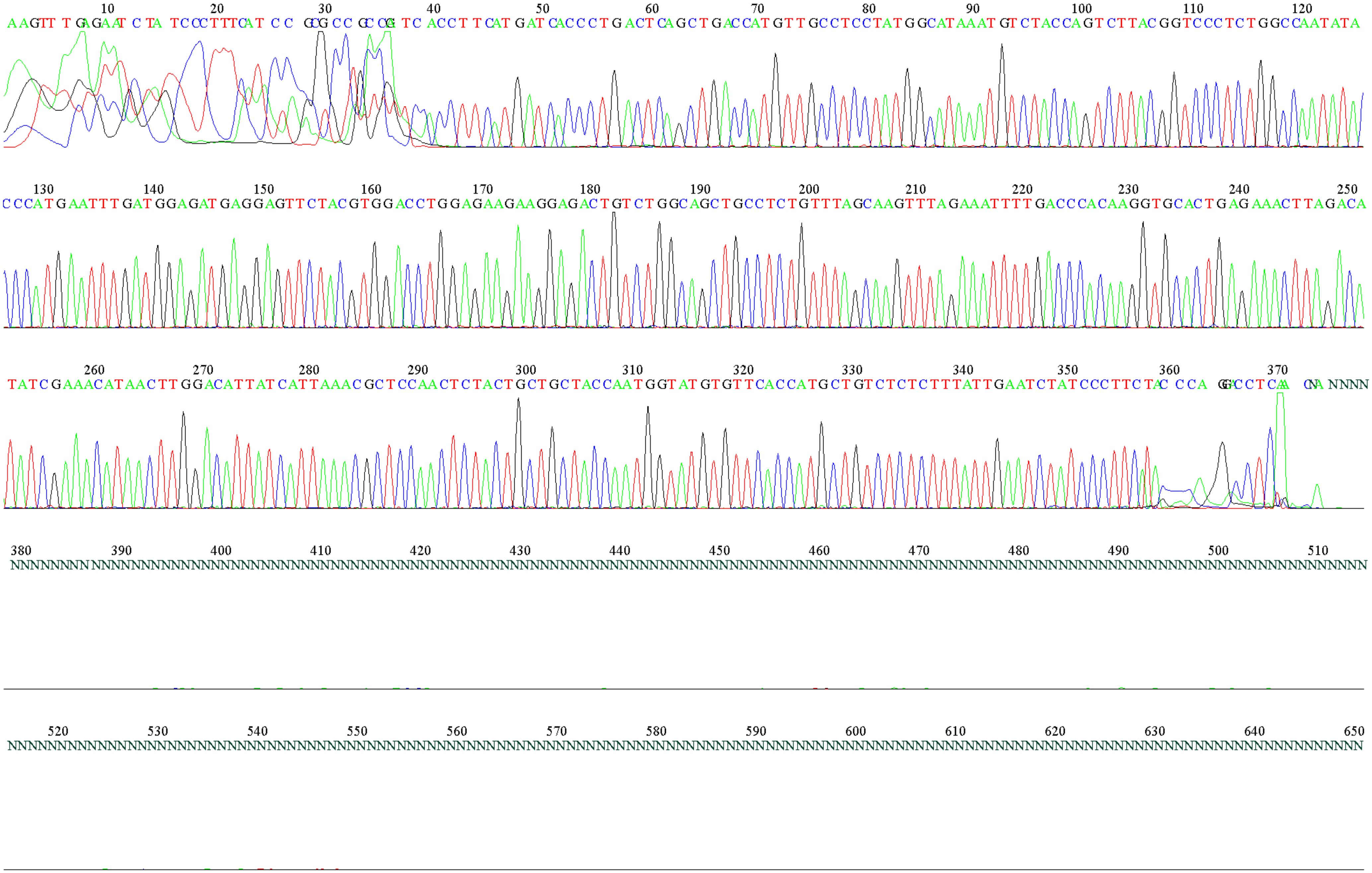

# DQA*02

MACROGEN
*Advancing through Genomics*

*File: 13EFBAA005-19.ab1*    *Run Ended: 2013/3/21 6:29:5*    *Signal G:3264 A:2553 C:2339 T:2734*

*Sample: 13EFBAA005-19_premix*    *Lane: 44*    *Base spacing: 15.09152*    *1359 bases in 16300 scans*    

10 20 30 40 50 60 70 80 90 100 110 120

AAAGTTA A TATCTATCCCT TCAT CCA GGCCT AAA TC ACCTTCATGCTCAACCCGACTCAGCTGACCATGTTGCCTCTTACGGCGTAAATGTCTACCAGTCTTACGGTCCCTCTGGCCAATATACC

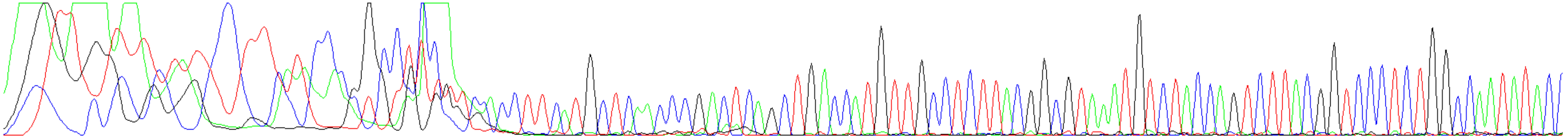

130 140 150 160 170 180 190 200 210 220 230 240 250

CATGAATTTGATGGAGATGAGCAGTTCTACGTGGACTTGGAGAAGAAGGAGACTGTCTGGCAGCTGCCTCTGTTTACCAAGTTTGCAAGTTTTGACCCACAAGGTGCACTGAGAAACTTAGACATA

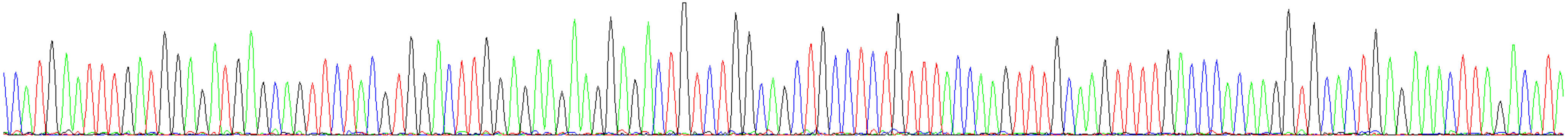

260 270 280 290 300 310 320 330 340 350 360 370

TCGAAACATAACTTGGACATTATCATTAAACGCTCCAACTCTACTGCTGCTACCAATGGTATGTGTTCACCATGCTGTCTCTCTTTATTGAATCTATCCCTTC TAC ACAGGCCTCA CANNNNN

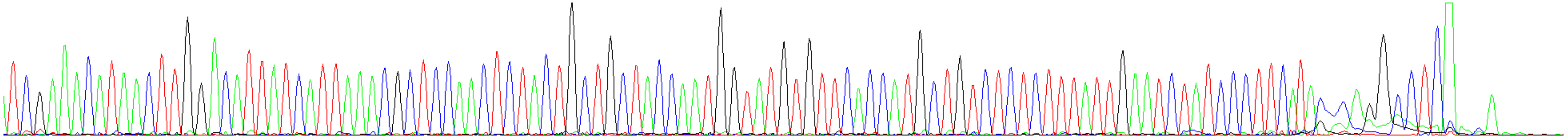

380 390 400 410 420 430 440 450 460 470 480 490 500

NNNNNNN NNNNNNNNNNNNNNNNNNNNNNNNNNNNNNNNNNNNNNNNNNNNNNNNNNNNNNNNNNNNNNNNNNNNNNNNNNNNNNNNNNNNNNNNNNNNNNNNNNNNNNNNNNNNNNNNNNNN



510 520 530 540 550 560 570 580 590 600 610 620

NNNNNNNNNNNNNNNNNNNNNNNNNNNNNNNNNNNNNNNNNNNNNNNNNNNNNNNNNNNNNNNNNNNNNNNNNNNNNNNNNNNNNNNNNNNNNNNNNNNNNNNNNNNNNNNNNNNNNNNNNNNN

# DQA*03

*File: 13EFBAA003-39.ab1    Run Ended: 2013/2/15 6:6:8    Signal G:5573 A:6148 C:5684 T:5644*

*Sample: 13EFBAA003-39_premix    Lane: 94    Base spacing: 15.1371565    1448 bases in 16300 scans    Page 1 of 2*

MACROGEN Advancing through Genomics

10 20 30 40 50 60 70 80 90 100 110 120

AAGTTATAATCCTGTCCTTCATCCACCGCCGACGTCACCTTCATGATCACCCTGACTCAGCTGACCATGTTGCCTCCTATGGCATAAATGTCTACCAGTCTTACGGTCCCTCTGGCCAATACAC

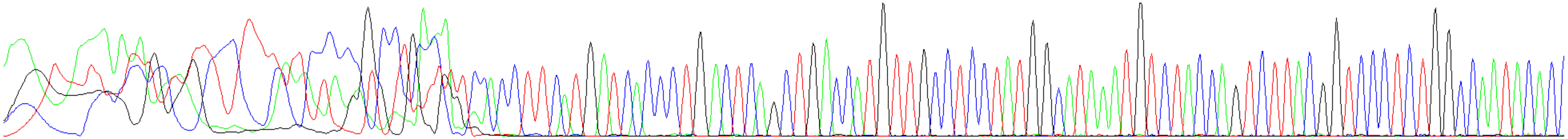

130 140 150 160 170 180 190 200 210 220 230 240 250

CCATGAATTTGATGGAGATGAGGAGTTCTACGTGGACCTGGAGAAGAAGGAGACTGTCTGGCAGCTGCCTCTGTTTAGCAAGTTTAGAAATTTTGACCCACAAGGTGCACTGAGAAACTTAGACAT

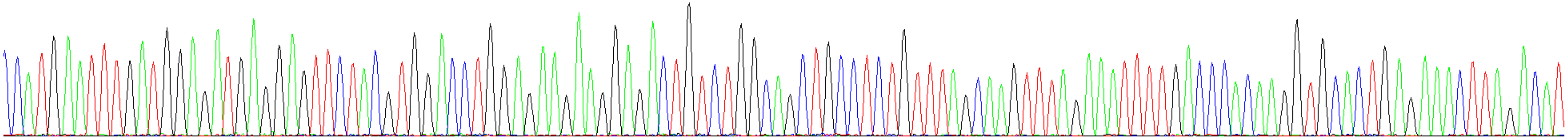

260 270 280 290 300 310 320 330 340 350 360 370

ATTGAAACATAACTTGGACATTATCATTAAACGCTCCAACTCTACTGCTGCTACCAATGGTATGTGTTCACCATGCTGTCTCTCTTTATTGAATCTATCCCTTCTACCCAGGGCCTCAAGAANNNN

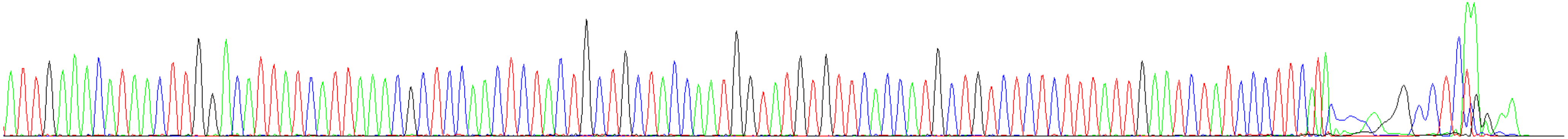

380 390 400 410 420 430 440 450 460 470 480 490 500 510

NNNNNNNNNNNNNNNNNNNNNNNNNNNNNNNNNNNNNNNNNNNNNNNNNNNNNNNNNNNNNNNNNNNNNNNNNNNNNNNNNNNNNNNNNNNNNNNNNNNNNNNNNNNNNNNNNNNNNNNNNNNNNNNNNNNNNNNN

520 530 540 550 560 570 580 590 600 610 620 630 640

NNNNNNNNNNNNNNNNNNNNNNNNNNNNNNNNNNNNNNNNNNNNNNNNNNNNNNNNNNNNNNNNNNNNNNNNNNNNNNNNNNNNNNNNNNNNNNNNNNNNNNNNNNNNNNNNNNNNNNNNNNNNNNNNNNNNNNNN
