# Supplementary material for: The major histocompatibility complex in Old World camelids and low polymorphism of its class II genes
Source: BMC Genomics. 2016 Mar 1;17:167. doi: 10.1186/s12864-016-2500-1 (PMC4774177; doi:10.1186/s12864-016-2500-1)

DQB-homozygote

File: 166CBAB002-5.ab1   *Run Ended: 2014/8/13 0:19:58*   *Signal G:1743 A:1281 C:1340 T:650*

Sample: 166CBAB002-5_premix   *Lane: 20*   *Base spacing: 14.114236*   *1000 bases in 16152 scans*

MACROGEN
Advancing through Genomics

10 20 30 40 50 60 70 80 90 100 110 120

GGG CGTT TA TG AGCAGG TGCG GCTCGC GCA CA CAC CGT CTAC ACCA GGAGGAGTACGT GCGCTTCGACAGCGACTGGGGGGAGCAGGGCGGGGAGTTCCGGGCGGTGACCGAGCTGGGGCGGCC

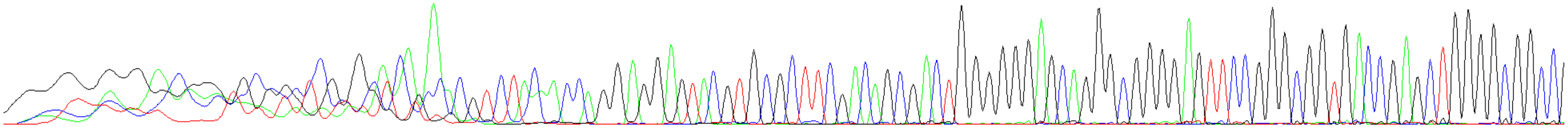

130 140 150 160 170 180 190 200 210 220 230 240 250

GGACGCCGAGTACTGGAACAGCCAGAAGGACGTCCTGGAGCGGAAGCGGGCCGAGCTGGACACGGTGTGCAGACACAACTAN CTGCAGCTCAANNN GNNNNNNNNNNNNNNNNNNNNNNNNNNNNNNN

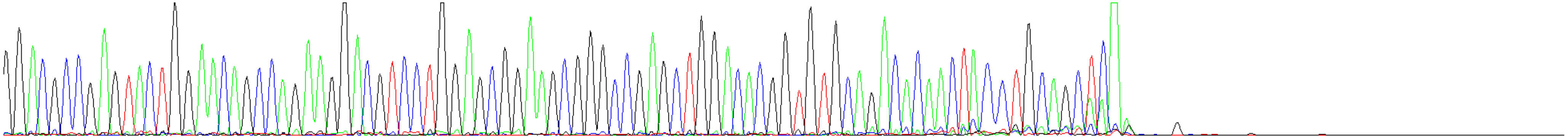

260 270 280 290 300 310 320 330 340 350 360 370 380 390

NNNNNNNNNNNNNNNNNNNNNNNNNNNNNNNNNNNNNNNNNNNNNNNNNNNNNNNNNNNNNNNNNNNNNNNNNNNNNNNNNNNNNNNNNNNNNNNNNNNNNNNNNNNNNNNNNNNNNNNNNNNNNNNNNNNNNNNNNNNNNN

400 410 420 430 440 450 460 470 480 490 500 510 520

NN N NNNNNNNNNNNNNNNNNNNNNNNNNNNNNNNNNNNNNNNNNNNNN NNNNNNNNNN N NNNNNNNNNNNNNNNNNNNNNNNNNNNNNNNNNNNNNNNNNNNNNNNNNNNNNNNNNNNNNNNNNNNNNNNNNNNNNNNNN

530 540 550 560 570 580 590 600 610 620 630 640 650 660

NNNNNNNNNNNNNNNNNNNNNNNNNNNNNNNNNNNNNNNNNNNNNNNNNNNNNNNNNNNNN NNNNNNNNNNNNNNNNNNNNNNNNNNNNNNNNNNNNNNNNNNNNNNNNNNNNNNNNNNNNNNNNNNNNN NNNNNNNNNN

DQB-heterozygote1

File: 166CBAB002-29.ab1 *Run Ended: 2014/8/13 0:19:58* *Signal G:8184 A:5923 C:7756 T:3148*

Sample: 166CBAB002-29_premix *Lane: 51* *Base spacing: -16.163063* *213 bases in 16302 scans*

MACROGEN
Advancing through Genomics

10 20 30 40 50 60 70 80 90 100 110 120 130

GGACGGTAGTGAGCGGGTGCGGCTCGTGACCAGAGCGTCTAAACCGGGAGGAGTACGTGCGCTTCGACAGCGACTGGGGGAGCACGGCGGGGACTACCGGCCGGTGACCGAGCTGGGGCGGCCGGACGCC

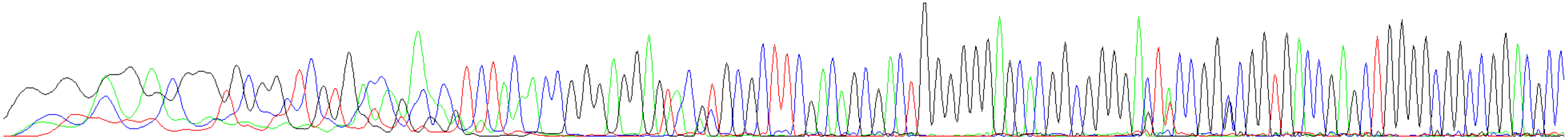

140 150 160 170 180 190 200 210

GAGTACTGGAACAGCCAGAAGGACGTCCTGGAGCAGACGCGGGCCGAGCTGGACACGGTGTGCAGACACAACTACCTGCAGC

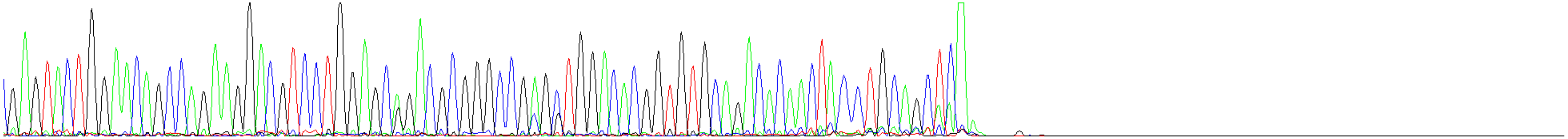

Supplement: Additional file 5: — Chromatograms of selected DQB sequences. (PDF 77 kb) [file 12864_2016_2500_MOESM5_ESM.pdf]
